# Optimizing care for children with difficult-to-treat and severe asthma through specialist paediatric asthma centres: expert practical experience and advice

**DOI:** 10.1186/s12887-024-04707-0

**Published:** 2024-03-27

**Authors:** M. W. Pijnenburg, S. Rubak, H. O. Skjerven, S. Verhulst, V. Elenius, C. Hugen, O. Jauhola, C. Kempeneers, E. Melén, T. Reier Nilsen, N. W. Rutjes, M. Ruotsalainen, H. Schaballie, A. M. Zwitserloot, M. Proesmans, M. J. Mäkelä

**Affiliations:** 1 Department of Pediatrics, Turku University Hospital, Turku, Finland; 2 Department of Pediatrics, Radboud University Medical Center, Nijmegen, The Netherlands; 3 Department of Allergology, Helsinki University Hospital, Helsinki, Finland; 4 Division of Respirology, Department of Pediatrics, University Hospital Liège, Liège, Belgium; 5 Department of Allergology, HUS, Helsinki University Hospital, Allergic Diseases and University of Helsinki, P.O. Box 160, FIN-00029 Helsinki, Finland; 6 Sachsska Children’s Hospital and Department of Clinical Science and Education, Södersjukhuset, Karolinska Institutet, Stockholm, Sweden; 7 Division of Paediatric and Adolescent Medicine, Oslo University Hospital, Oslo, Norway; 8 Faculty of Medicine, Institute of Clinical Medicine, University of Oslo, Oslo, Norway; 9 Department of Paediatrics, Division of Respiratory Medicine and Allergology, Erasmus University Medical Centre – Sophia Children’s Hospital, Rotterdam, The Netherlands; 10 Department of Pediatrics, University Hospital Leuven, Leuven, Belgium; 11 Department of Development and Regeneration, Catholic University of Leuven, Leuven, Belgium; 12 The Norwegian Olympic Sports Centre, Oslo, Norway; 13 Department of Sports Medicine, Oslo Sport Trauma Research Centre, Oslo, Norway; 14 Danish Center of Pediatric Pulmonology and Allergology, Department of Pediatrics and Adolescent Medicine, Department of Clinical Medicine, University Hospital of Aarhus, Aarhus University, Aarhus, Denmark; 15 Department of Pediatrics, Kuopio University Hospital, Kuopio, Finland; 16 Department of Pediatric Pulmonology and Allergy, Emma Children’s Hospital, Amsterdam UMC, University of Amsterdam, Amsterdam, The Netherlands; 17 Pediatric Pulmonology, Pediatric Department, Ghent University Hospital, Ghent, Belgium; 18 Laboratory of Experimental Medicine and Pediatrics, University of Antwerp, Antwerp, Belgium; 19 Department of Pediatrics, Antwerp University Hospital, Antwerp, Belgium; 20 Beatrix Children’s Hospital Department of Pediatric Pulmonology and Pediatric Allergy, University of Groningen, University Medical Center Groningen, Groningen, The Netherlands; 21 University of Groningen, University Medical Center Groningen, Groningen Research Institute for Asthma and COPD (GRIAC), Groningen, The Netherlands; 22 Skin and Allergy Hospital, Meilahdentie 2, Helsinki, Finland

**Keywords:** Difficult-to-treat asthma, Severe asthma, Children, Asthma care, Biologics, Asthma nurses

## Abstract

Severe asthma in children carries an unacceptable treatment burden, yet its rarity means clinical experience in treating it is limited, even among specialists. Practical guidance is needed to support clinical decision-making to optimize treatment for children with this condition.

This modified Delphi convened 16 paediatric pulmonologists and allergologists from northern Europe, all experienced in treating children with severe asthma. Informed by interviews with stakeholders involved in the care of children with severe asthma (including paediatricians, nurses and carers), and an analysis of European guidelines, the experts built a consensus focused on the gaps in existing guidance. Explored were considerations for optimizing care for patients needing biologic treatment, and for selecting home or hospital delivery of biologics. This consensus is aimed at clinicians in specialist centres, as well as general paediatricians, paediatric allergologists and paediatric pulmonologists who refer children with the most severe asthma to specialist care. Consensus is based on expert opinion and is intended for use alongside published guidelines.

Our discussions revealed three key facets to optimizing care. Firstly, early asthma detection in children presenting with wheezing and/or dyspnoea is vital, with a low threshold for referral from primary to specialist care. Secondly, children who may need biologics should be referred to and managed by specialist paediatric asthma centres; we define principles for the specialist team members, tests, and expertise necessary at such centres, as well as guidance on when homecare biologics delivery is and is not appropriate. Thirdly, shared decision-making is essential at all stages of the patient’s journey: clear, concise treatment plans are vital for patient/carer self-management, and structured processes for transition from paediatric to adult services are valuable. The experts identified the potential for specialist paediatric asthma nurses to play a significant role in facilitating multidisciplinary working.

Through this project is agreed a framework of practical advice to optimize the care of children with severe asthma. We encourage clinicians and policymakers to implement this practical advice to enhance patient care.

**Supplementary Information:**

The online version contains supplementary material available at 10.1186/s12887-024-04707-0.

## Background

Asthma is common in childhood, and most children achieve good symptom control with low-to-medium doses of inhaled corticosteroids. However, for some, symptoms remain problematic despite high treatment doses [1, 2]. Asthma can be described as ‘difficult to treat’ when it is uncontrolled despite medium- or high-dose inhaled corticosteroids with a second controller and/or with maintenance systemic corticosteroids; or when high-dose treatment is needed to control symptoms and reduce the risk of exacerbations [3]. Difficult-to-treat asthma relates to modifiable factors such as poor adherence, incorrect inhaler technique, comorbidities or adverse exposures [1, 4]. After addressing modifiable factors, children whose disease remains poorly controlled on high doses of medication form a heterogeneous group considered to have ‘severe’ asthma phenotypes [4], and are eligible for step-up therapies. These include long-acting muscarinic antagonists, oral corticosteroids, and most recently, biologics targeting immunoglobulin (Ig)E, interleukin (IL)-4Rα, IL-5, and IL-13 [1, 5].

Severe asthma in children is a rare disease, accounting for less than 5% of all childhood asthma cases [4, 6], yet it can cause frequent asthma attacks and hospital admissions, and its mortality is unacceptably high [4]. Furthermore, although biologics have been available for managing asthma in adults and adolescents for almost two decades, research on their efficacy and safety in children has lagged [7]. Accordingly, clinical experience in managing severe childhood asthma is limited, even among specialists.

This project convened paediatric pulmonologists and allergologists from northern Europe. Its initial aim was to give consensus-based practical advice on best practices to support clinical decision-making in two areas: first, considerations around biologic treatment for children aged 6–18 years with severe asthma; and second, on providing optimal care for these patients. We also considered how to disseminate these considerations throughout healthcare settings. As the project progressed, these objectives evolved with its emerging findings, and we refocused our aims on the considerations for selecting home or hospital delivery of biologics, as practical advice on this topic was found to be lacking.

This consensus is aimed at clinicians in specialist centres, as well as general paediatricians, paediatric allergologists and paediatric pulmonologists who refer children with the most severe asthma to specialist care. Consensus is based on expert opinion and is intended for use alongside published guidelines.

## Methods

This was a modified Delphi, completed between June 2022 and May 2023. Delphi is a useful approach for gaining consensus when data are limited and expert opinion is needed to shape clinical judgements [8–10]. This two-round Delphi was done using online surveys.

### Definition of consensus

Responses to survey statements were recorded using a 5-point Likert scale (1 [‘strongly disagree’] to 5 [‘strongly agree’]), similar to other consensus projects [9]. Experts could explain their responses to support statement modification between rounds.

Consensus was defined a priori when ≥ 75% of responses scored 4 or 5 (‘agree’ or ‘strongly agree’). Partial consensus occurred if some – but not all – parts of a multi-part survey item reached consensus.

### Participants

The expert voting group comprised paediatric pulmonologists or allergologists working in a variety of secondary or tertiary settings across northern Europe (Sweden, Norway, the Netherlands, Finland, Denmark or Belgium; Table 1).

**Table 1:** Composition of the expert voting group

Experts (*N* = 16)	n (%)
Clinical role	
Paediatric pulmonologist	11 (69)
Paediatric allergologist	5 (31)
Country of practice	
Belgium	4 (25)
Finland	4 (25)
The Netherlands	4 (25)
Norway	2 (13)
Sweden	1 (6)
Denmark	1 (6)

These clinicians were invited based on their practical expertise, publishing records, national and regional standing, and interest. A subset of five experts formed a steering committee.

The sponsor abstained from discussions and had no input on the surveys or consensus. The views reported are the authors’ alone.

### Stakeholder interviews, survey development and voting

The steering committee agreed the project’s scope, target patients and topics for consideration, and was informed by a gap analysis of guidelines [3, 5, 11–16]; this project focused on the gaps.

To explore these gaps, further inform the survey and reflect the needs and experience of patients, carers and relevant clinicians, we gathered insights from a stakeholder group. This comprised three secondary care/private paediatricians, two specialist paediatric asthma nurses, two pulmonologists specializing in adult care, one representative of a patient organization, and one carer of a child with severe asthma. Individual exploratory 60-min Zoom interviews were conducted in English by an independent facilitator and an observer, assisted by a translator when necessary. Recordings and contemporaneous notes were analysed to identify themes.

Using these inputs, draft statements were developed by the Delphi facilitator, refined and agreed by the steering committee, then voted on by the entire expert group. The steering committee reviewed the Round 1 results and developed the Round 2 survey, focusing on topics not at consensus. A virtual meeting elaborating results allowed the experts to contextualize their opinions; no further voting occurred.

## Results

### Delphi results

**Fig. 1:**
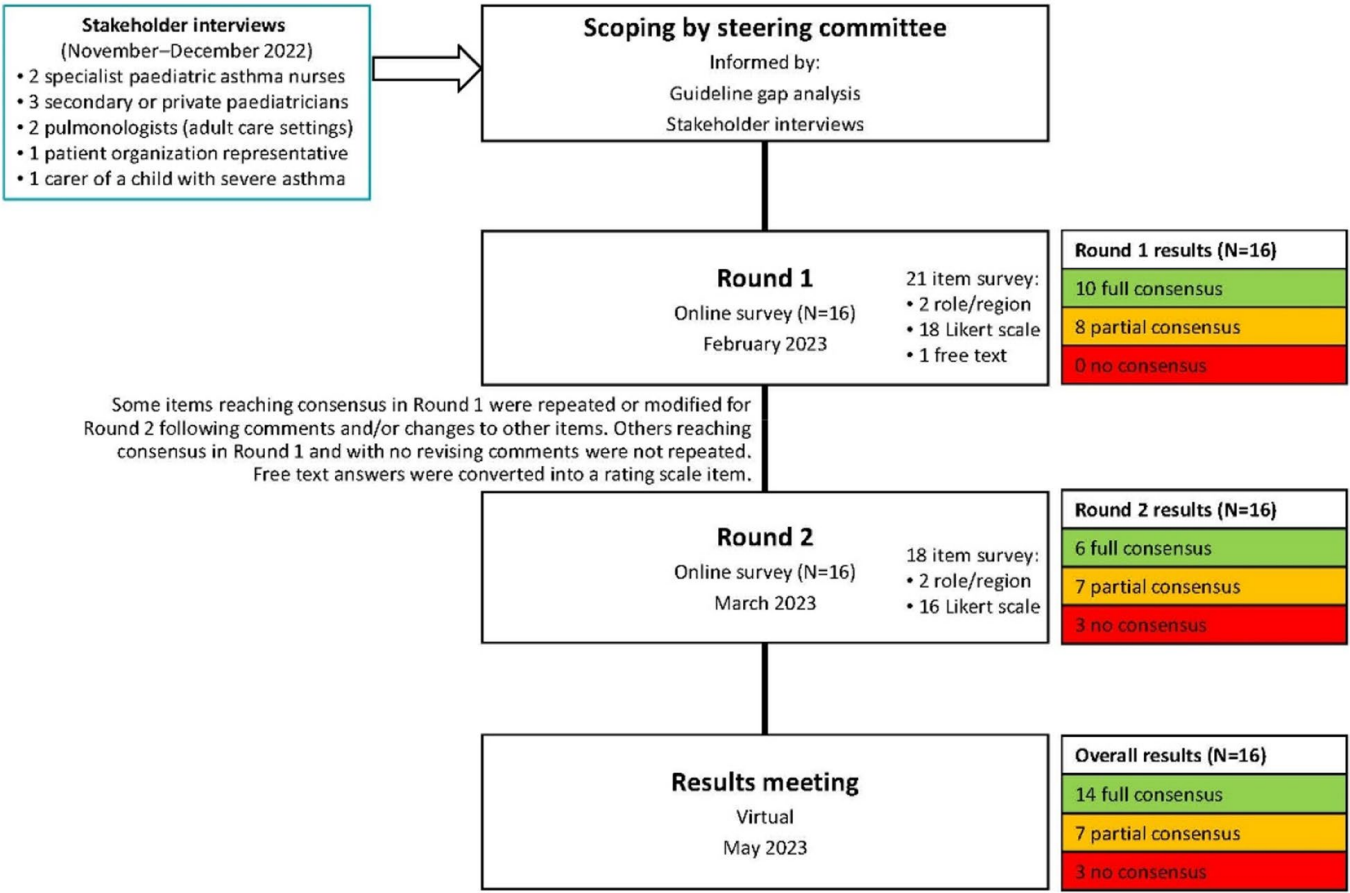
Project flow chart

After both rounds, 14 items were at full consensus, 7 at partial consensus and 3 at no consensus (Fig. 1). All data can be found in the Supplementary data file.

The survey evolved to have several overarching themes. Notably, following the direction of the discussions informed by the gap analysis, the initial goal to explore the considerations around biologic treatment for children aged 6–18 years with severe asthma was refocused on the considerations for selecting home or hospital delivery of biologics. This was because practical advice on this topic was found to be lacking, and an educational need, while detailed recommendations on biologics have already been published by Global Initiative for Asthma (GINA) [5] and European Academy of Allergy & Clinical Immunology (EAACI) [14].

#### Diagnostic considerations in primary care

Stakeholder interviews highlighted that accessing clinical teams with experience of managing children with severe or difficult-to-treat asthma can be difficult, because such children are so rare that few experts have extensive clinical experience of treating them. The stakeholders noted that lacking access to such specialists is a barrier to patients’ optimal holistic care. There was also a concern that such children may reach the most specialist centres only after experiencing a severe exacerbation.

**Fig. 2:**
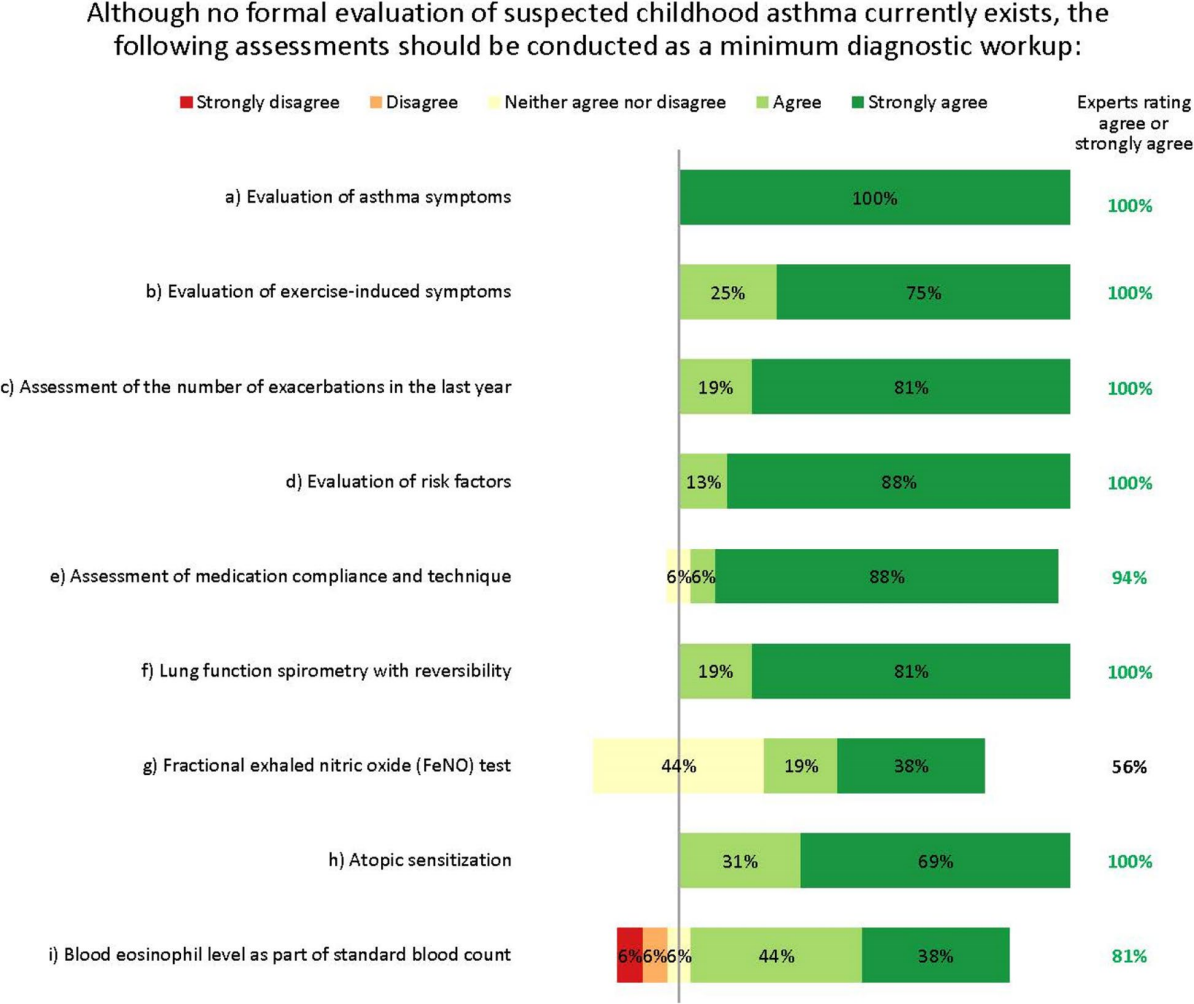
Minimum diagnostic workup for childhood asthma. Numbers are shown in green for those statements at consensus

The expert group therefore agreed that detecting asthma early in children presenting with respiratory symptoms (e.g. wheeze, dyspnoea) – regardless of severity – is vital. They agreed that the minimum diagnostic workup for suspected asthma should evaluate signs and symptoms from both upper and lower airways, lung function, exacerbations, risk factors, medication compliance, atopic sensitization and blood eosinophil levels (Fig. 2). The experts agreed on the importance of assessing atopic sensitization and blood eosinophil levels, but advocated a stepwise testing approach, reserving these assessments until after other tests have been done. Fractional exhaled nitric oxide (FeNO) testing did not reach consensus as part of the initial minimum diagnostic workup, but was considered useful for differential diagnosis or as an additional diagnostic tool, and possibly later when choosing which biologic to use.

#### When to refer to a specialist paediatrician or specialist paediatric asthma centre

The clinical settings in which these tests should be done and diagnosis made was explored. Stakeholder interviews and steering committee discussions revealed that the differentiation between primary, secondary and tertiary care for children with asthma is misleading, as some specialist paediatric asthma centres do not sit in tertiary settings, and some patients may be managed in tertiary settings that are not specialist paediatric asthma centres. We therefore differentiate between specialist care (secondary and tertiary care) and care in specialist paediatric asthma centres (which have the highest level of expertise and experience).

**Fig. 3:**
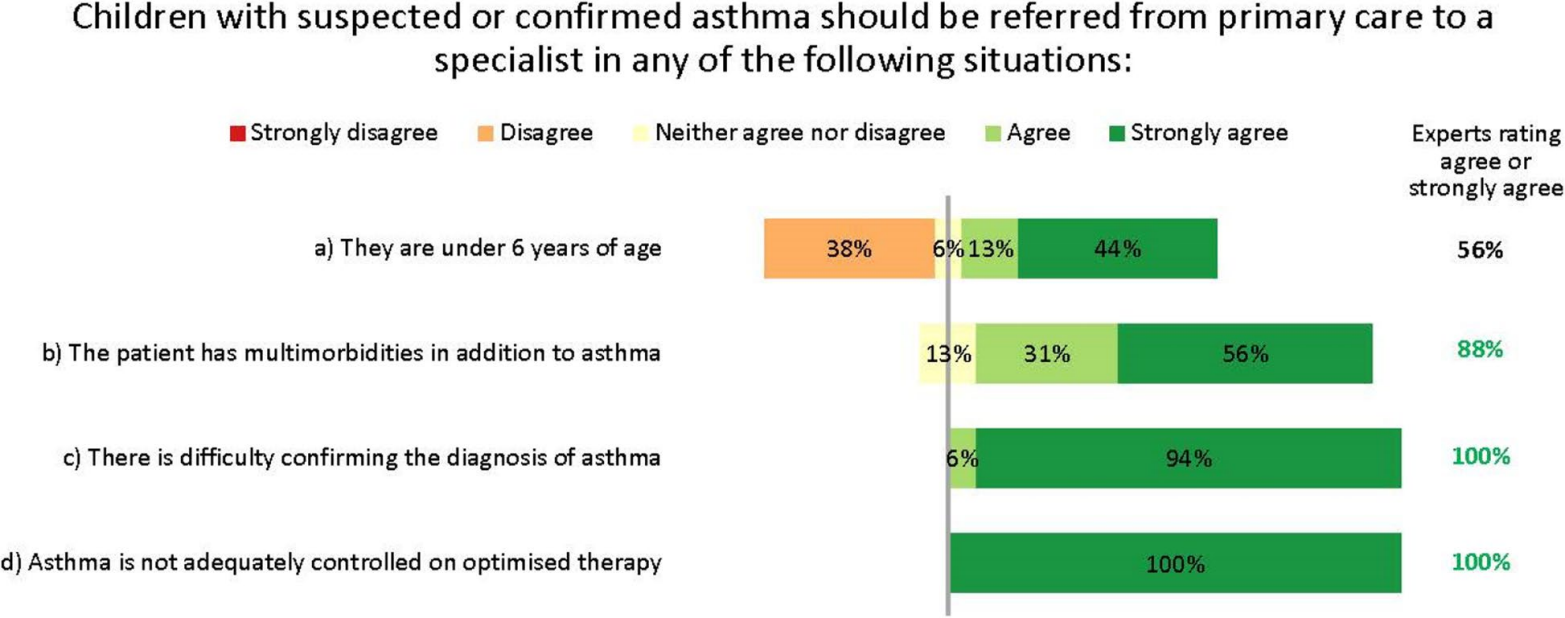
Referral from primary to specialist care. Numbers are shown in green for those statements at consensus

The experts suggested that the adequate tests, as available, should be done in primary care, with referral to specialist care or even a specialist paediatric asthma centre for further testing and confirmation of an asthma diagnosis in case of doubt or complicating factors (Fig. 3). However, they recognize a ‘one-size-fits-all’ approach would be undesirable. For example, referral to a specialist is unnecessary if a correct diagnosis and control can be achieved in primary or secondary care. Conversely, limitations of resources or expertise may prevent some general practitioners (GPs) from diagnosing accurately without specialist assistance. Indeed, the availability of diagnostic tests varies considerably by setting and region.

In discussions, the experts strongly agreed that the threshold for referral to specialist care should be low, to reduce the risk that children with potentially serious disease are misdiagnosed or undertreated. Notably, the experts disagreed that children aged under 6 years need automatic referral. In many cases, a decision to refer will depend less on age than on comorbidities and severity, as well as the facilities and expertise available in primary care. Further, viral-induced (‘preschool’) wheeze is so common in children of this age that automatic specialist care is almost always unwarranted in the absence of other risk factors. The experts unanimously agreed that children with difficult-to-treat or severe asthma who may need biologic treatment should be referred to and managed by specialist paediatric asthma centres. This referral may be direct from primary or secondary care, depending on local practices.

#### Appropriate diagnostic tests for specialist paediatric asthma centres

**Fig. 4:**
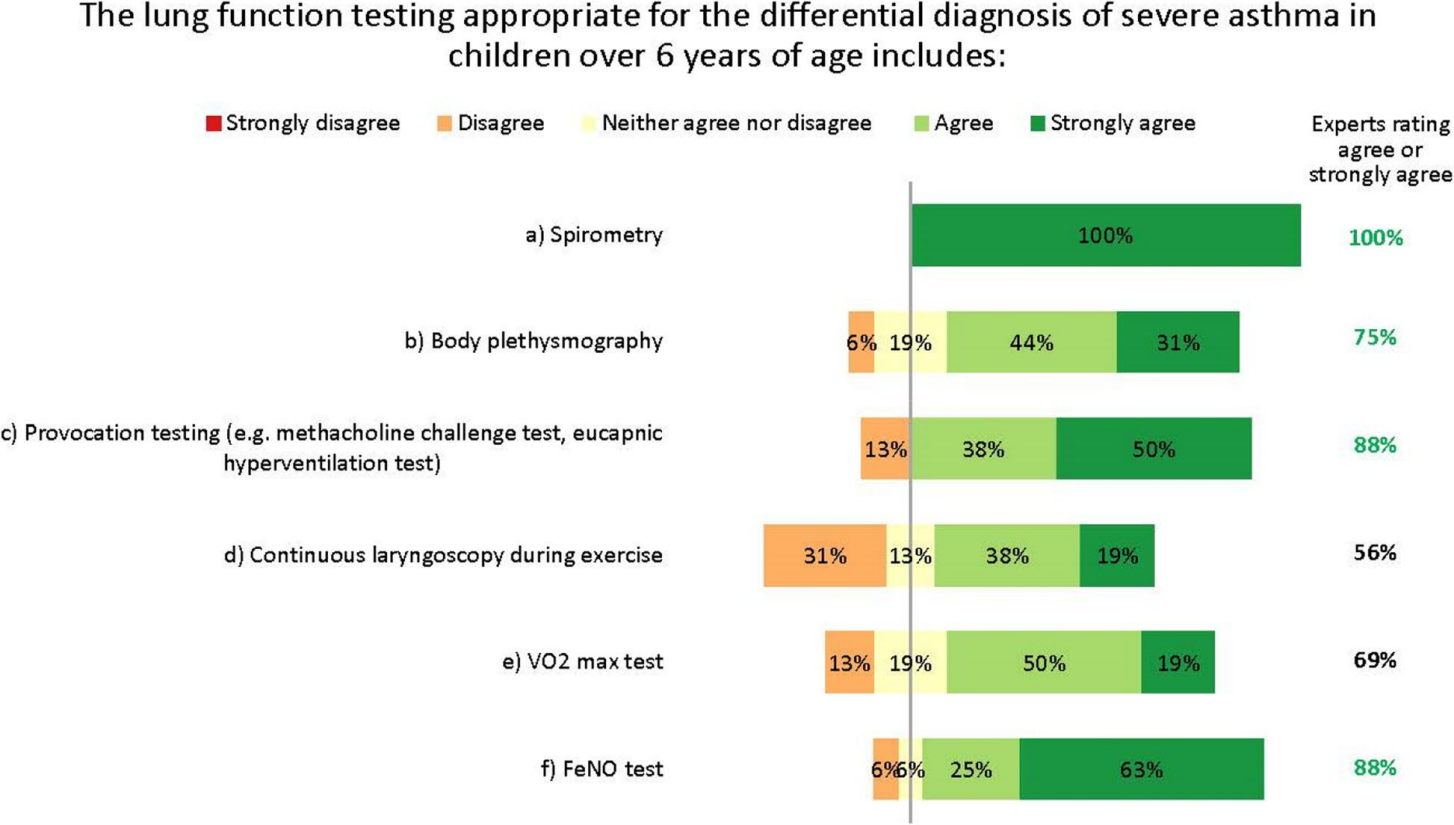
Lung function testing for diagnosis. Numbers are shown in green for those statements at consensus

The experts did not fully agree which lung function tests are appropriate for the differential diagnosis of severe asthma in children aged over 6 years. The differences in opinion largely result from regional differences in practices and resources, as well as the wide variation in patients’ characteristics, meaning general recommendations are difficult to give. A unanimous consensus was reached for spirometry with bronchodilator response testing, and most experts agreed on body plethysmography, provocation testing and FeNO testing. Others suggested oscillometry in cases where spirometry is insufficient, such as for younger children [17]. However, no agreement was reached on continuous laryngoscopy during exercise or on volume of oxygen (VO_2_) max testing (Fig. 4). Further, the experts acknowledge that many more tests than these also exist for monitoring asthma in children, such as the lung clearance index and infant lung function test. These are not yet widespread in northern Europe and are not suitable for all children or centres, but the experts suggest they may be helpful in some clinical scenarios, as recently discussed by Pijnenberg, et al. [18].

### Practical considerations for specialist paediatric asthma centres

**Fig. 5:**
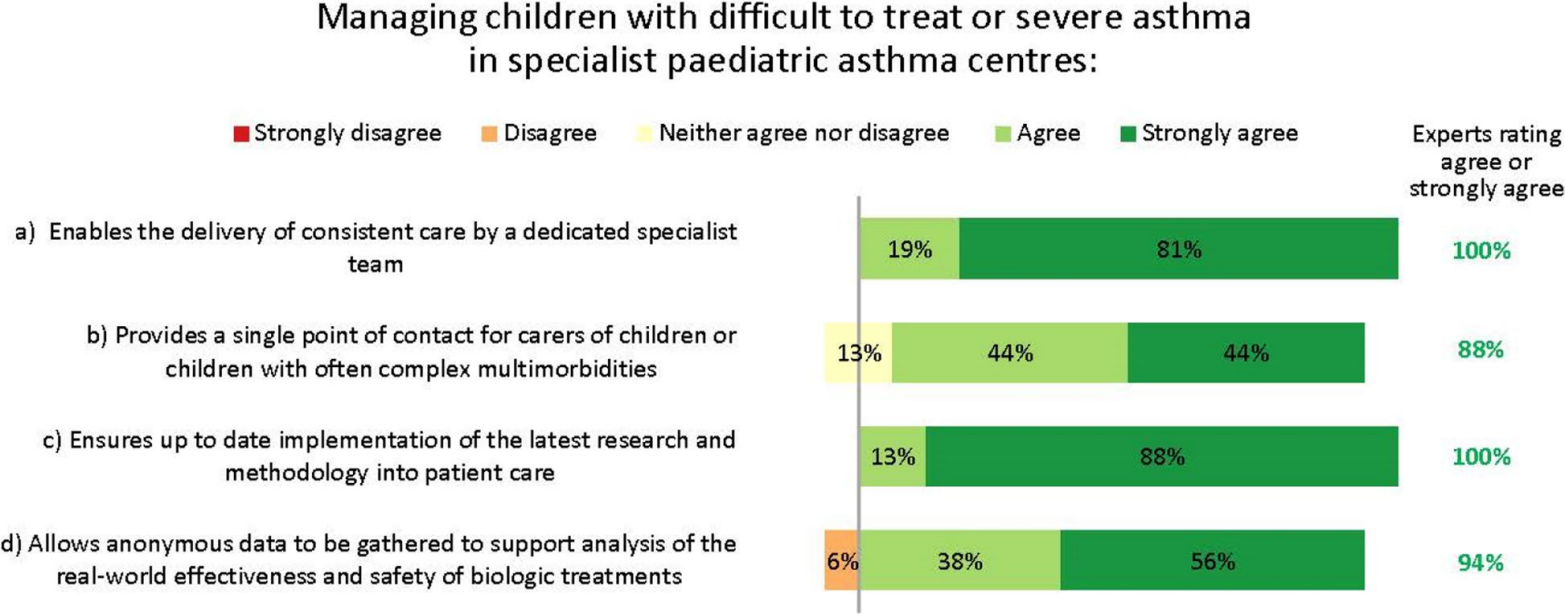
Importance of specialist paediatric asthma centres. Numbers are shown in green for those statements at consensus

The experts saw multiple reasons for managing children with difficult-to-treat or severe asthma who may need biologic treatment in specialist paediatric asthma centres (Fig. 5).

#### Essential facilities and tests

**Fig. 6:**
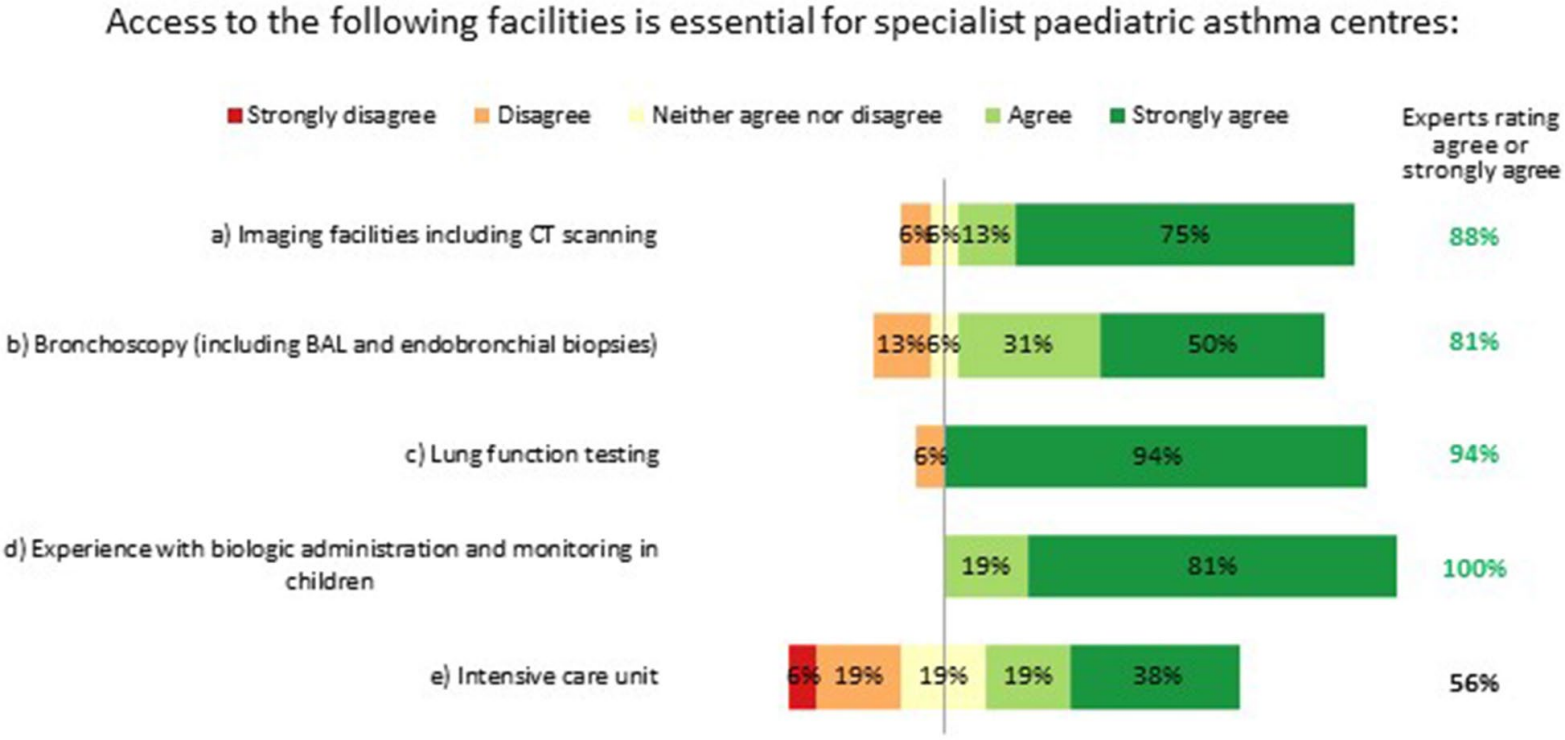
Essential facilities for specialist paediatric asthma centres. Numbers are shown in green for those statements at consensus. BAL: bronchoalveolar lavage; CT: computed tomography

Specialist paediatric asthma centres must have access to imaging facilities, bronchoscopy and lung function testing (spirometry, body plethysmography, provocation testing and FeNO testing), and have experience with biologic administration and monitoring in children. Having an intensive care unit in the same centre was not considered essential, but having access to such facilities elsewhere was deemed desirable (Fig. 6).

#### Multidisciplinary staff and working practices

The experts stressed the importance of having care delivered by a multidisciplinary team working at the specialist paediatric asthma centre. In the interest of improving the standards of holistic care, the experts suggest that such teams should include the perspectives of a diverse range of specialists. For example, the following likely provide essential insights: a paediatric pulmonologist/allergologist or paediatrician; a paediatric pulmonary nurse or specialist asthma nurse; a pulmonary function analyst (or similar); a medical social worker or paediatric psychologist; and a physiotherapist with paediatric experience. Access to a dietician, a geneticist or an immunologist may be valuable should a patient’s individual circumstances warrant this. We suggest that the best composition of multidisciplinary teams will need tailoring within the constraints of existing healthcare systems and local practices, and warrants closer research.

**Fig. 7:**
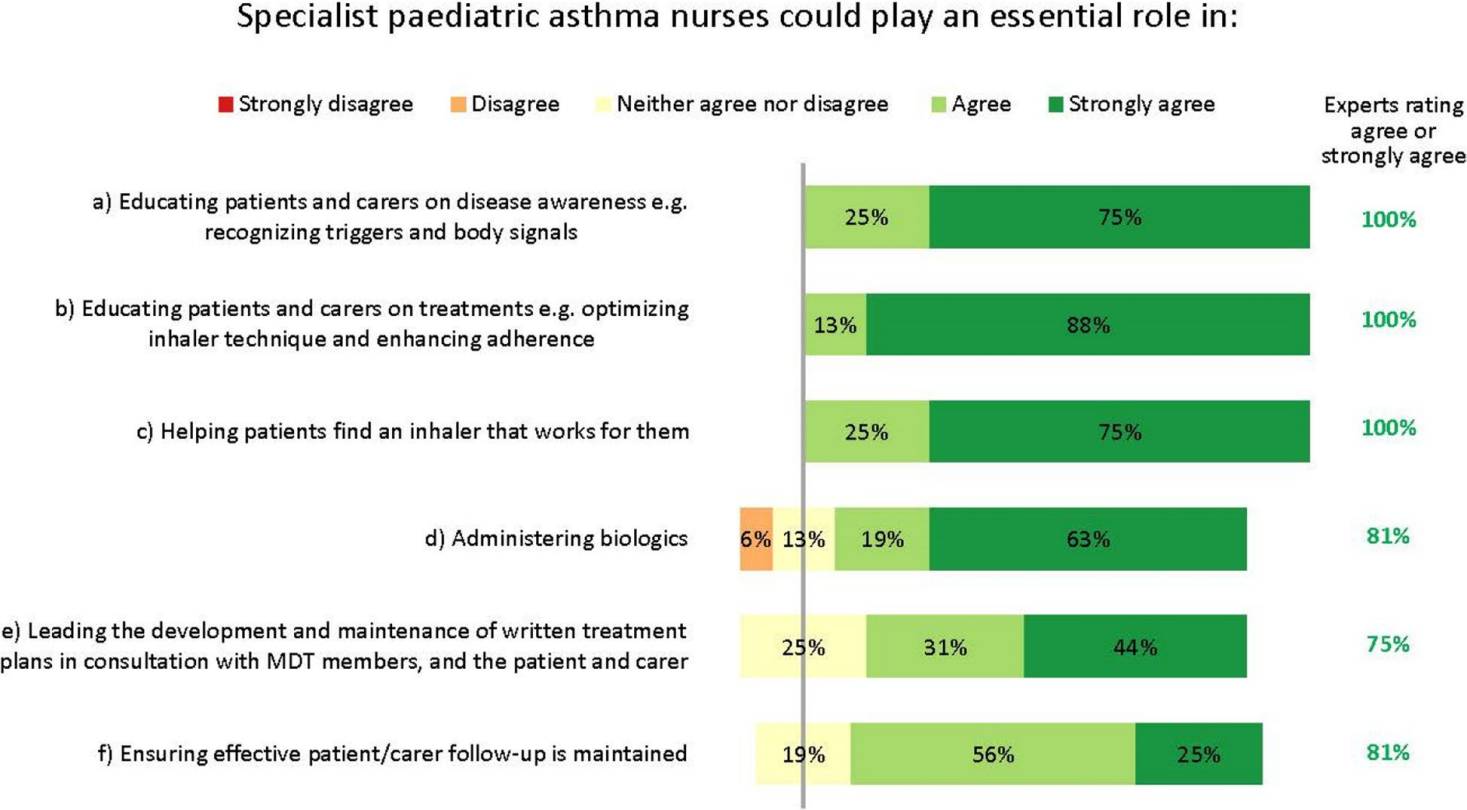
Roles of specialist paediatric asthma nurses. Numbers are shown in green for those statements at consensus. MDT: multidisciplinary team

Specialist paediatric asthma nurses have an influential role in coordinating and optimizing a patient’s care as part of the multidisciplinary team (Fig. 7). Interestingly, nurses’ importance and potential were recognized even though several experts work in settings where nurses play no key role at present. While recognizing the constraints that healthcare settings face, the experts unanimously agreed many essential facets of a nurse’s role (Fig. 7). Specialist – or possibly general – nurses could help administer biologics, according to local practices.

The experts also agreed specialist nurses could lead the development and maintenance of written treatment plans, in consultation with the multidisciplinary team, the patient and their carer (Fig. 7). These plans should be concise and clear, and contain top-line information on the patient’s treatment goals, medications and emergency contacts, and advice on when and how to contact the medical team.

### Shared decision-making and communication across clinical settings

The experts almost unanimously agreed that patients and their carers should be at the centre of decision-making on all aspects of their care and treatment; this requires joined-up management. However, stakeholder interviews revealed that collaboration between primary, specialist and specialist paediatric asthma care providers is inconsistent and uncommon, and from a carer/patient perspective, this can be a barrier to effective care.

The experts reached consensus that specialist paediatric asthma centres should keep primary care providers – and other services involved in a patient’s care – updated on treatment and management needs, especially for complex cases. However, the practicality of doing this was a concern, with some experts highlighting operational difficulties such as the administrative burden of communicating with multiple settings. Others suggested that electronic health records are sufficient to provide effective cross-setting communication, and that these are usually accessible to GPs. Further, almost all experts agreed that occasionally inviting representatives of other care settings to consultations is appropriate, but that resourcing challenges may preclude this.

Some experts suggested that specialist paediatric asthma nurses, given their oversight of treatment plans, could help bridge the gap between the specialist paediatric asthma centre and primary or secondary health providers.

#### Transition to adult services

**Fig. 8:**
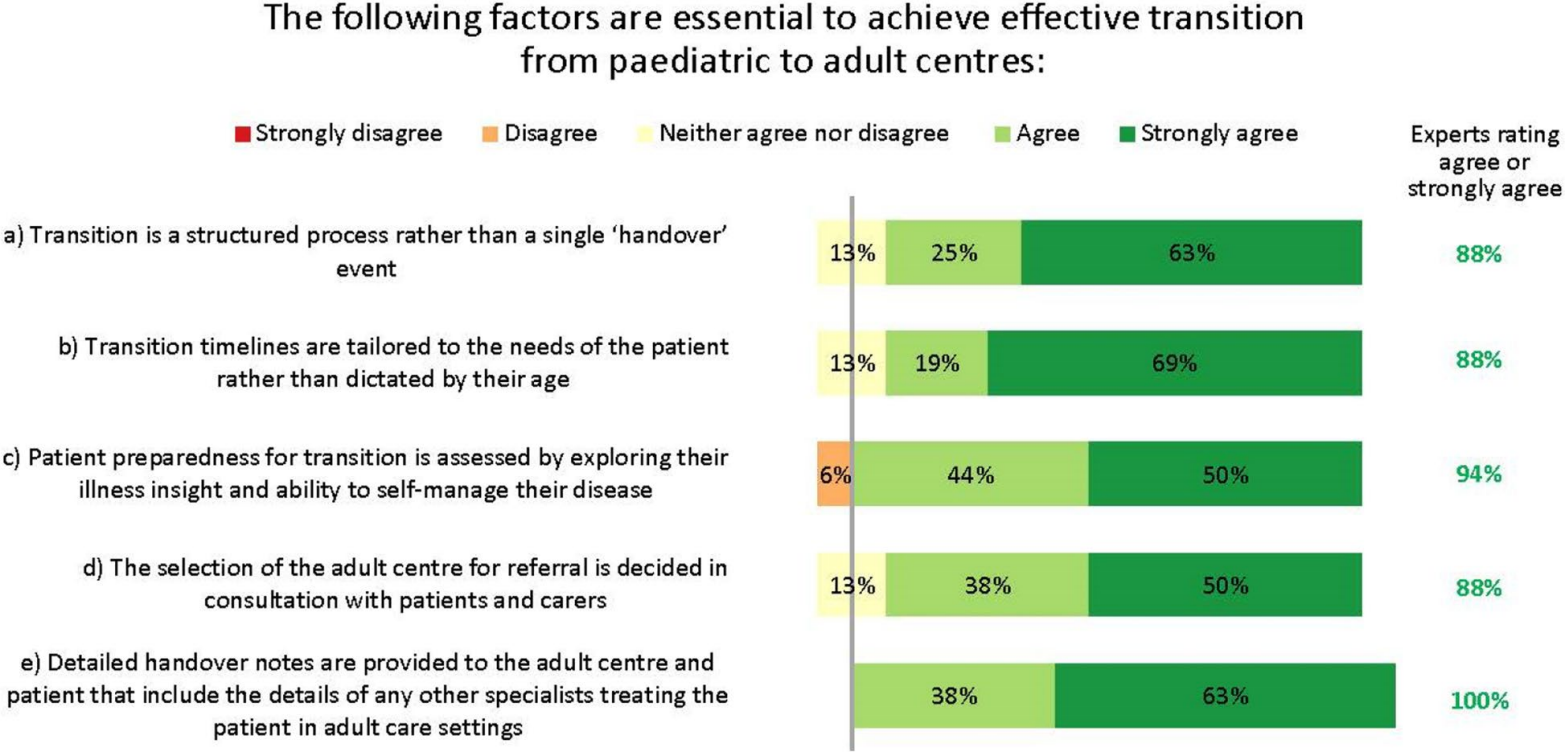
Factors needed for effective paediatric–adult services transition. Numbers are shown in green for those statements at consensus

The GINA guidelines state that the process of transition for paediatric to adult care should support the adolescent in gaining greater autonomy and responsibility for their own health and wellbeing [5]. However, despite its importance, stakeholder interviews revealed that this transition is inconsistent and can be poor [19]. Transition varies by country and clinical setting, and usually occurs at ages 15–18 years. Transition generally proceeds via an electronic referral only, although sometimes patients are handed over following telephone briefings or joint consultations between the paediatric and adult physicians, and often their primary care providers. The experts agreed several factors essential for achieving an effective transition (Fig. 8).

Almost all clinical stakeholders interviewed said they would like to improve transition, and some had tried but encountered barriers, such as limited resources and complexity of scheduling joint consultations between paediatric and adult centres. However, many voting experts (63%) felt that having at least one joint consultation between the paediatric and adult specialists before formal transition would be desirable.

One major complexity associated with transition is that many patients have multimorbidities. In the paediatric setting, these are often managed by paediatricians, but in adult settings, multiple clinicians may be involved: patients report this to be overwhelming and confusing. Clinicians felt that patients prioritized the conditions of greatest impact to them and deprioritized others.

#### Homecare delivery of biologics

The experts unanimously agreed that when choosing a biologic, caregivers should consider clinical and practical factors and patient/carer preferences. However, in reality, cost and reimbursement also play a role in treatment decisions.

**Fig. 9:**
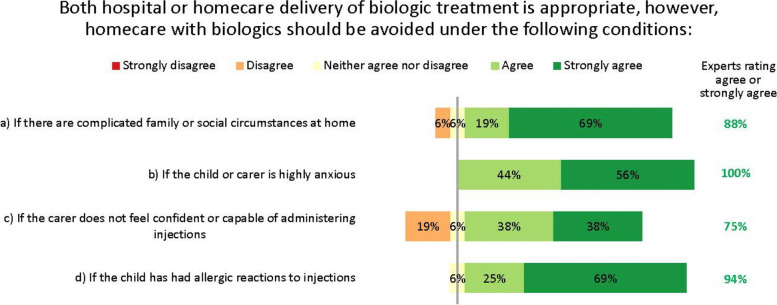
Circumstances in which homecare biologics delivery is inappropriate. Numbers are shown in green for those statements at consensus

The experts agreed that for children, both hospital and homecare delivery of biologics can be appropriate, but in alignment with other Delphi groups considering adult patients, recognised that this is not suitable for all patients [20]. They therefore suggested some guidance on when to avoid homecare. Home delivery of biologics should be avoided for patients with complicated family or social circumstances, severe anxiety, lack of confidence in injection ability, or prior allergies to injections (Fig. 9). Despite the consensus, some suggested the injection confidence barrier could be overcome if injections were given by nurses instead of the patient or carer at the patient’s home, school or nearest healthcare centre.

The experts did not reach consensus on whether home spirometry may enable effective home delivery of biologics in patients with poor symptom perception. Most (56%) recognized that home spirometry can be useful to help patients recognize their symptoms, but that it can be unreliable in children because of a lack of cooperation or proficiency. Others suggested that if a patient’s symptom perception remains poor despite using home spirometry, then this would indicate biologics to be given at a clinical centre, not at home.

#### Research priorities for biologics

Agreed areas for further research on biologics relate mostly to treatment escalation/de-escalation, biomarkers and head-to-head comparisons between agents (Table 2). Where the experts did not reach consensus, it was because sufficient evidence already exists and not because the topic is of low priority.

**Table 2:** Consensus on priority research topics in biologics in children

Topic	Experts rating strongly agree or agree, %
Comparison of effectiveness of different biologics in children	100
How to select the most appropriate biologic for each patient	100
When and how to switch biologic treatment	100
When and how to stop biologic treatment	100
Biomarkers to support clinical decision-making	100
Collection and analysis of registry data on real-world asthma management	88
When and how to start biologic treatment	75

## Discussion

This project convened northern European specialists in managing children with severe and difficult-to-treat asthma. While rare, these patients carry a substantial treatment burden [1]. The advent of biologic treatment has provided much-needed treatment, but questions remain unanswered about the practicalities of optimal clinical management. As a result of this project, we have agreed a framework of practical advice to optimize the care of children with difficult-to-treat and severe asthma. We encourage clinicians and policymakers to implement this practical advice to enhance patient care.

### Key suggestions

**Fig. 10:**
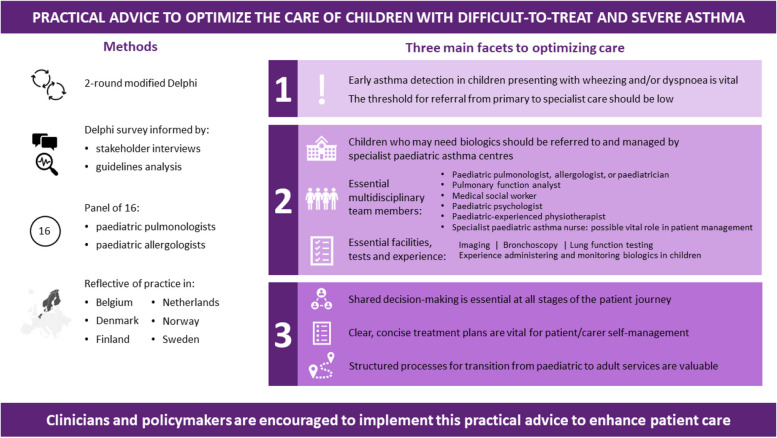
Practical advice to optimize the care of children with difficult-to-treat and severe asthma

Our experts’ key recommendations centre on three facets, which are summarized in Fig. 10 for quick reference: 1) early detection of asthma in children presenting with wheezing and/or dyspnoea is vital and the threshold for referral from primary to specialist care should be low; 2) children who may need biologics should be managed by specialist paediatric asthma centres, for which we have defined facilities, staff and responsibilities; and 3) shared decision-making and inter-setting communication is vital at all stages of the patient journey, up to and including transition to adult services.

#### Early detection of asthma and referral to specialist care

In some cases, diagnosing asthma in children can be done in primary care, without referring to secondary or tertiary care. However, in some countries including Belgium, primary care providers lack access to the essential diagnostic tests to make such a diagnosis. Further, some guidelines recommend that diagnostic tests for children should be done by a specialist [16]. Since the differential diagnosis can be complicated by comorbidities or non-specific symptoms, and asthma may be severe or refractory, some cases warrant referral to centres with more centralized expertise, i.e. specialist paediatric asthma centres. Crucially, the threshold for such referrals should be low [21]. This aligns with recommendations from other groups [1], including those of the UK National Working Group of asthma experts, who further suggest that timely consultation after referral is vital [20]. Our interviews and Delphi showed the triggers for referral to specialist care are unified, and align with recognized treatment-escalation criteria such as those in the GINA guidelines [5].

Interestingly, while many experts favoured its use, we did not reach consensus that FeNO testing should be mandatory for the minimum diagnostic workup for children with suspected childhood asthma, mostly because it is inconsistently available and reimbursed throughout Europe. However, its use may become more widespread now that FeNO testing is recommended by the ERS as part of the diagnostic workup for children aged 5–16 years [12]. Nevertheless, most experts agreed FeNO is useful as an add-on test for differential diagnosis, endotyping, or for more advanced diagnostics in specialist settings, and that together with measuring total/specific IgE, could be most helpful after diagnosis has been made; for example when choosing which biologic to use. These suggestions reflect what is possible and practicable within standard clinical care [12].

#### Specialist paediatric asthma centres

Our experts felt that providing care in specialist paediatric asthma centres could have many benefits for children with severe asthma. Indeed, the need for, and value of such centres has been shown in multiple studies [21]. Such benefits include providing patients with access to consistent care by a dedicated specialist team (something our interviewees suggested is currently lacking); giving patients a single point of contact, which may be especially important for those with complex comorbidities; and delivering optimal care informed by the latest research and clinical advances.

These benefits may be particularly acute for patients receiving biologic treatment for the most severe asthma. As our discussions and uncertainties evolved, it became clear that, while detailed clinical recommendations on biologics use have been published by GINA [5] and EAACI [14], there is little *practical* advice on the holistic aspects of biologics use, including the considerations for selecting home or hospital delivery of biologics, although recent Delphi projects have begun to address this [20]. Reflecting uncertainties in the literature, the experts did not reach consensus on whether home spirometry has a role in providing optimal treatment for patients on biologics, with some experts concerned about its reliability in children. For example, a 2006 retrospective study in 36 children found poor concordance between asthma severity scores and home spirometry indices among children with severe asthma [22]; conversely, a 2019 interventional study in 77 children reported improved asthma control among those who used home spirometry combined with a self-management app compared with those using conventional treatment [23]. Other studies have similarly conflicting results [24, 25]. Despite this uncertainty, over half our experts felt that home spirometry could be valuable in some situations. Postulated benefits include the potential to help patients better recognize their symptoms, and the ability to monitor symptoms in a patient’s home environment, not an artificial clinic environment. This could inform the decision on whether the biologic should be given at a clinical centre or at home, and this potential application has prompted research into improving the reliability of this technique in children [23].

#### Shared decision-making and inter-setting communication

Because severe and difficult-to-treat asthma is complex, heterogenous, and often occurs with comorbidities [26], it cannot be treated in isolation: asthma care must form part of a holistic care plan in conjunction with other specialties and specialist centres, and patients themselves. Care must be integrated vertically between primary, secondary and specialist paediatric asthma settings, as well as horizontally among health, education and social services, acknowledging the impact of constraints in each area upon the others [21]. The importance of shared care is underscored many times throughout this initiative and by other researchers [1, 20, 21], but the reality is often different: a source of frustration for clinicians and patients [27].

An area where improving shared care is particularly needed is around the longitudinal transition between paediatric and adult care. In recognition of this need, the EAACI published specific guidance on this topic in 2020 [28], and our discussions complement their recommendations. Transition needs advanced planning and should not be a one-time event, but barriers to successful transition are legion, including a lack of standardized processes, difficulties with advanced planning, and suboptimal communication [13, 29]. GINA guidelines state that the process of transition should support the young person in gaining greater autonomy and responsibility for their own health and wellbeing [5]. This is an important yet mostly overlooked consideration, with only the Finnish and British Thoracic Society (BTS) guidelines addressing the subject in detail [13, 16]. Our experts’ recommendations are in line with those of the BTS, and include the need for structure, individual tailoring and patient centricity [13], and recognise the essential role of primary care providers.

### Further findings

One interesting theme from our consensus is that the experts believe specialist paediatric asthma nurses could help in achieving optimal care. In the Netherlands, for example, nurses have an important role in managing the care of children with asthma. Similarly, the Finnish Care Guideline recommends that specialist nurses be involved in the treatment and care of asthma in children under school age, and for those aged < 12 years if local practices allow [16]. Nevertheless, many of our experts work in settings where nurses are not available at all, and others that do have them explained they are not effectively used to deliver paediatric asthma care.

Enhancing nurses’ existing roles to include facilitation and care oversight could improve cross-specialty and cross-setting communication. For example, by overseeing treatment plans, performing diagnostic tests and coordinating care de-escalation/escalation when appropriate, nurses could form vital bridges among all specialists involved in a patient’s care. Also, by assisting patients to receive their biologic injections at home, rather than in clinics, they could help relieve the pressure on clinic times and improve patients’ perceptions of care. This is supported by a qualitative international study in adults with severe asthma, published in 2022, which showed that the benefits of home administration of biologics usually outweigh the inconvenience and side effects: a view echoed by our experts [30].

Several studies have shown that these types of nurse-led activity can improve the outcomes of children with asthma [31, 32], as well as with other chronic conditions [33–35]. Those experts whose clinics employ nurses report that they are indispensable for helping patients manage their own asthma, for example by educating them on correct inhaler technique and avoiding exacerbating factors. Indeed, a Delphi consensus project from Canada supports this assertion, recognising the critical role nurses can play in patients’ education, empowerment and ongoing management [36]. We therefore suggest nurses may help manage the burgeoning costs of biologics, by ensuring patients only receive expensive biologic drugs when all other modifiable factors have been addressed [18]. Cost-effectiveness analyses are therefore warranted.

Coordinated, nurse-led communication could also improve the longitudinal transition from paediatric to adult services, which, as we have seen, is often haphazard or lacking. Indeed, having an identified coordinator who supports the young person throughout transition is recommended by the BTS, and nurses could fill this role [13].

### Strengths and limitations

This study has limitations, mostly relating to the size and composition of our expert and stakeholder groups. When scoping this project, the steering committee discussed whether to include GPs as part of the expert group. It was felt that since the project’s focus was on children with severe asthma, who are almost universally managed by specialists in the countries we represent, GPs were not included. However, we of course recognise that GPs are experts in recognising patients needing referral, thus referral needs were discussed in that context. We particularly appreciate the essential role that GPs play in managing transitional care for adolescents, and indeed, this was acknowledged during discussions. Nevertheless, our key focus was on the considerations for providing specialist attention beyond that available in primary care, and our expert group reflected this need.

Overall, few stakeholders with experience of biologics for children with difficult-to-treat or severe asthma were able to participate, citing time limitations, lack of relevant expertise with such patients and conflicts of interest. However, those who did participate all practise across a small number of northern European countries, and this focus became one of this project’s key strengths: we were well placed to highlight the similarities and differences between these countries.

Disappointingly, we found no psychologists or adolescent patients willing to be interviewed, so their views are absent. This also means that the opinions are skewed in favour of treating physicians and may overlook some considerations important for patients’ holistic care. In terms of experts, the number of specialist paediatric asthma centres across the countries is low, and experts from these centres in some (but notably not all) countries were precluded from participating in the Delphi by conflicts of interest.

### Future clinical and research implications

Throughout this project, the experts recognized that there is a disconnect between the ideal standard of care described in the consensus and what is feasible within the constraints of existing healthcare models. For example, the ability of primary care providers to perform diagnostic tests – even those noted here as being essential before onward referral – varies greatly among countries and settings, and depends heavily on local practices, expertise, funding and resources [16]. This disconnect is well understood and has inspired a call for a worldwide charter for asthma in children to better implement best practice recommendations [37]. Similarly, while our consensus defines the essential capabilities and personnel needed for specialist asthma centres, in alignment with suggestions from other countries [38, 39], in practice, the provision of these is limited by the same constraints. We suggest that investing in specialist nurses is a desirable aim for policymakers and healthcare planners. Their activities may help alleviate many of the bottlenecks and frustrations that exist within existing healthcare frameworks, improving the outcomes for patients with severe and difficult-to-treat asthma without the need for unrealistic structural changes to existing healthcare models.

## Conclusions

Specialist asthma centres are valuable for treating children with severe asthma, and the threshold for referral from primary to specialist care should be low, to optimize their diagnosis, treatment and quality of care. Especially, children who may need biologics should be managed by these specialist centres, for which we have defined facilities, staff and responsibilities. Lastly, shared decision-making and inter-setting communication is vital at all stages of the patient journey, up to and including transition to adult services.

This consensus reflects optimal care in an ‘ideal world’, and we encourage clinicians and policymakers in each country to consider how they could implement our suggestions to bring the reality closer to the ideal. We propose that investing in specialist nurses could overcome many difficulties resulting from poor cross-specialty and cross-setting communication, and could ease the burden on overstretched settings without necessitating unattainable structural changes.

### Supplementary Information

**Supplementary Material 1.**

## Data Availability

All data generated or analysed during this study are included in this published article and available as supplementary information.

## Abbreviations

BTS: British Thoracic Society
EAACI: European Academy of Allergy & Clinical Immunology
FeNO: Fractional exhaled nitric oxide
GINA: Global Initiative for Asthma
GP: General practitioner
