# Supplementary material for: Optimizing care for children with difficult-to-treat and severe asthma through specialist paediatric asthma centres: expert practical experience and advice
Source: BMC Pediatr. 2024 Mar 27;24:218. doi: 10.1186/s12887-024-04707-0 (PMC10967048; doi:10.1186/s12887-024-04707-0)
Supplement: Supplementary file 1 — Supplementary Material 1. [file 12887_2024_4707_MOESM1_ESM.pdf]

Optimizing care for children with difficult-to-treat and severe asthma through specialist paediatric asthma centres: expert practical experience and advice

Delphi Results

16. May. 2023

Initial asthma presentation and diagnosis

Initial asthma presentation and diagnosis: Item 3 R2

Although no formal evaluation of suspected childhood asthma currently exists, the following assessments should be conducted as a minimum diagnostic workup:

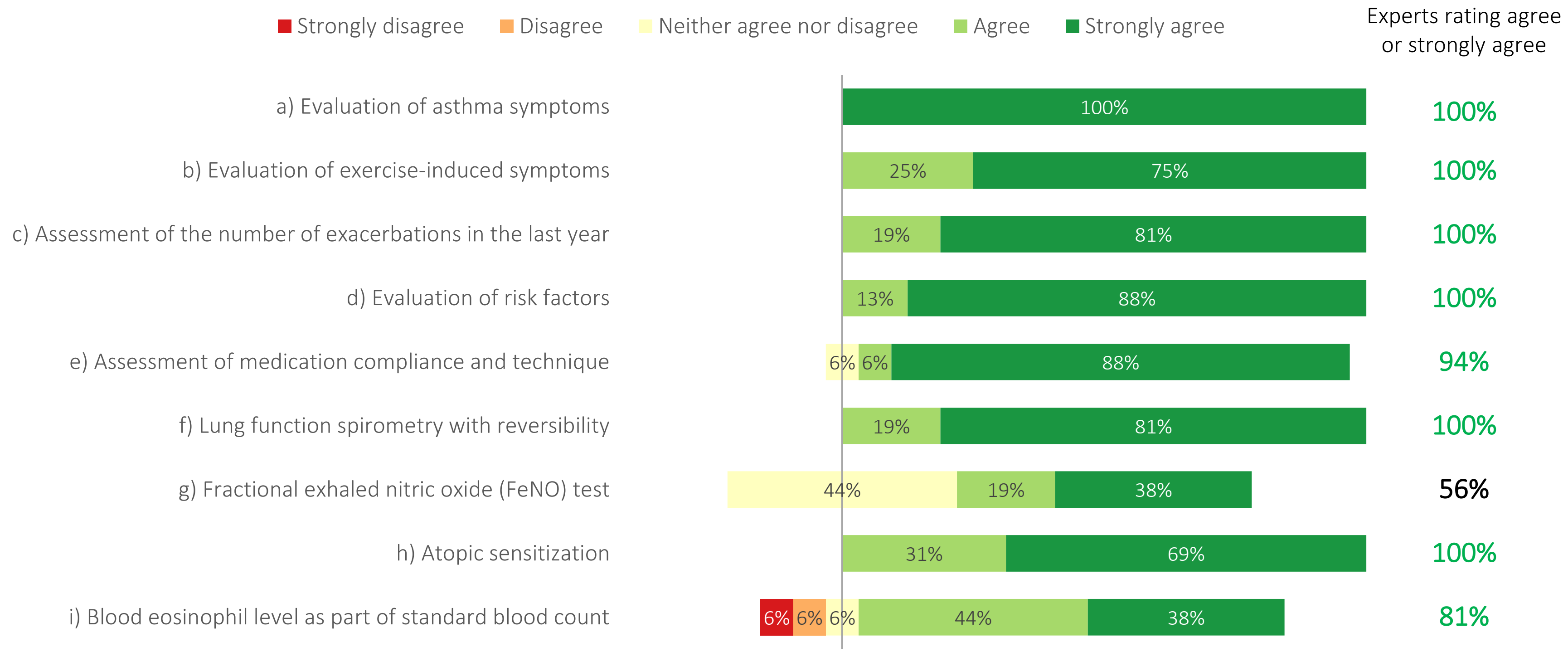

- g+h+i) not necessarily during the first round of assessments
- lab test is invasive in children, and blood eosinophil can help in management once asthma is diagnosed, but is not mandatory for first asthma diagnosis
- Oscillometry if the technique for spirometry is insufficient
- b) with history, not all patients need an exercise test, d) with history and potential further investigations if strongly suggestive (eg 24h ph measurement for reflux or PSG for OSAS)
- Spiro depending on age. FENO depending on availability/funding
- FeNo may be elevated in just allergic rhinitis, it's a good add, but not mandatory

Initial asthma presentation and diagnosis: Item 4 R2

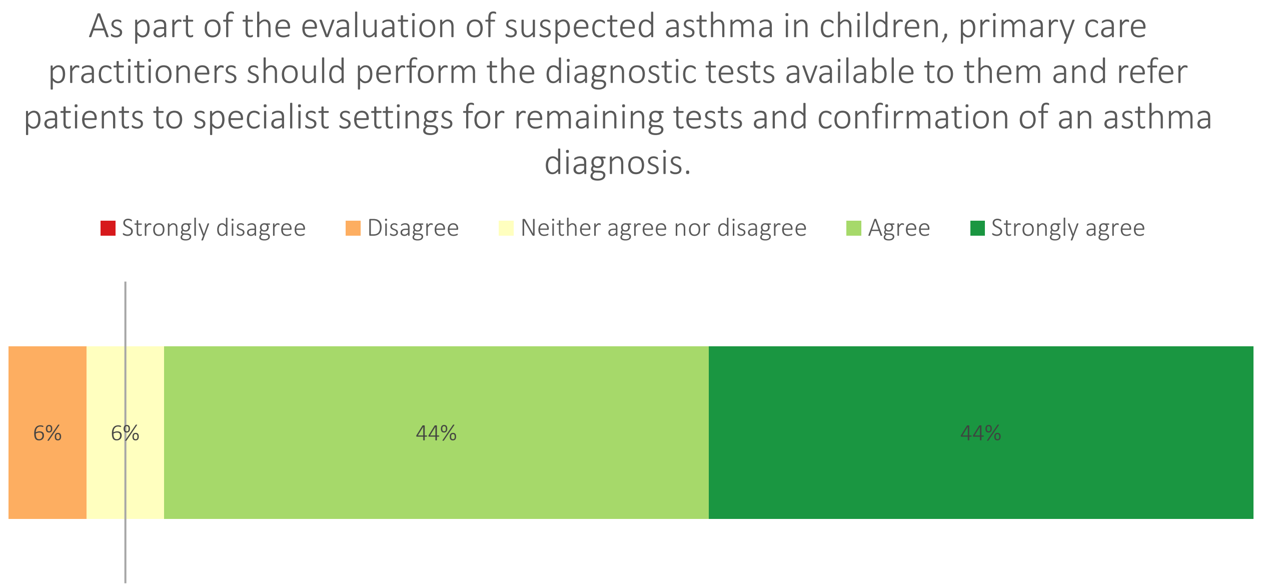

% rating agree or strongly agree	
R1	R2
75	88

- Agree when child is to be treated for longer time with ICS
- over 12 years yes, 6-12 mainly tertiary care
- spirometry in children needs experience and level of expertise to be performed correctly; atopy work-up can be performed in primary care, but not necessary for referral
- pediatric pulmonary function is not available in primary care in Belgium, it can be available in secondary care. If this is meant by 'specialist settings', then I agree. Not all patients with asthma symptoms need to be referred to tertiary care/asthma specialist center.
- in primary care mainly good clinical assessment a to e
- IF they have spirometry available then it should be used to evaluate presence of reversible air flow limitation. However in our country most primary care practitioners do not have spirometry available or are able to use it for children in the lower age ranges. It has already been demonstrated that if spirometry is not used there is a huge overdiagnosis and over treatment of children with respiratory symptoms. So I do strongly agree spirometry is used, but in daily practice I think availability is lacking.
- But if the GP has provided positive diagnostic tests for asthma, he/she does not have to refer for further confirmation, but may set the diagnosis him-/herself. Only in doubt, the child should be referred. But it must be totally clear to the GP, what is needed to set the diagnosis -
- most GPs will not have availability of F and G. they should refer if diagnostic uncertainty or uncontrolled asthma despite step 2 treatment.
- in small children yes. Adolescent, not complicated cases can be diagnosed in primary care

Initial asthma presentation and diagnosis: Item 5 R2

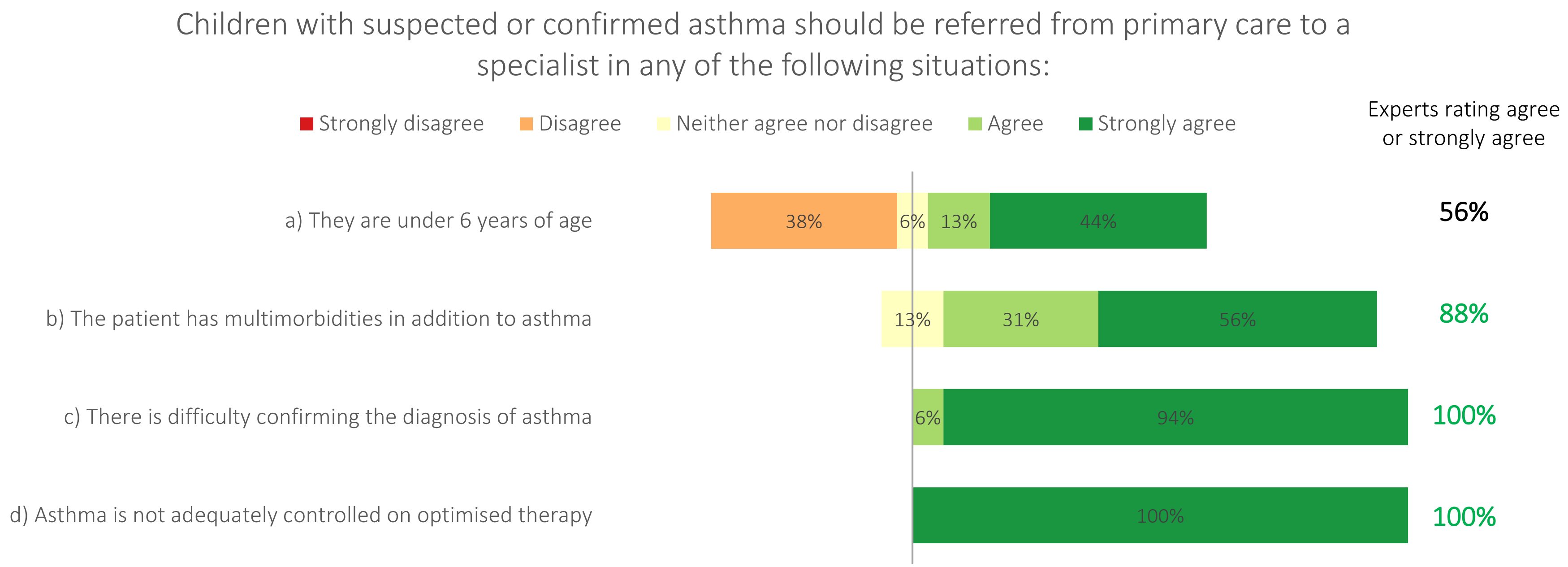

- a) Very young children < 2 years should be referred not all < 6 yrs. b) Depending on what morbidities are
- In our medical care system in Belgium, all children with asthma should see a specialist at least once a year, and at diagnosis, to confirm diagnosis and follow the treatment, with in-between visits that can be done by primary care or specialist, according to the ability of the primary care, accessibility of the specialist, and severity of the disease
- for children under 6 years with suspected asthma with intermittent wheeze, there is less added value for a specialist center, as they can not perform a reliable pulmonary function. Viral induced wheeze/preschool wheeze is so common that they can not all be followed by specialist center. I think all children above 6 with suspected asthma should be referred to a specialist (secondary care - pediatrician with access to pediatric PFT) for at least one PFT
- This really depends on how primary care is arranged. In my experience asthma is overdiagnosed in younger children in the primary care setting and there usually is not enough time for a full evaluation that also includes evaluation and treatment of co-morbidities. To me there should be a low threshold for referral to a general pediatrician unless primary care is organized differently. In addition I have seen problems with follow up of patients in the primary care setting.

Children with difficult-to-treat and severe asthma

Children with difficult-to-treat and severe asthma: Item 6 R1

Specialist asthma centres and staffing

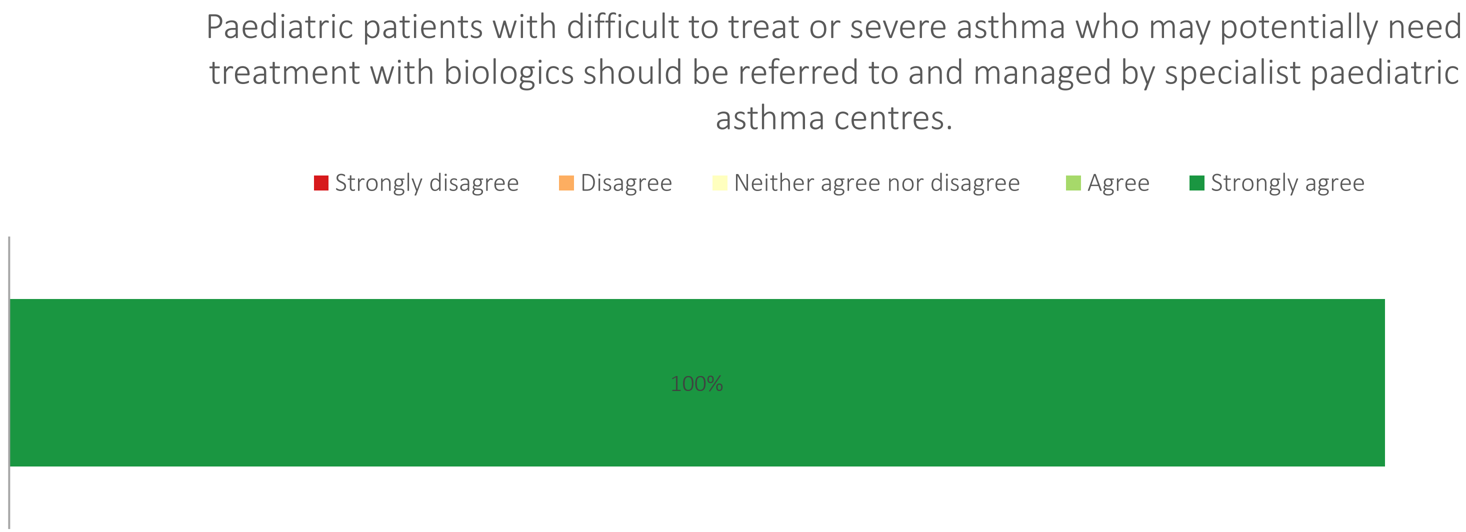

PLEASE ADD INFORMATION TO HELP EXPLAIN YOUR ANSWER

- This is so rare, that experience needs to centralized
- To a specialist in paediatric asthma, not necessarily a centre
- Only very few children with asthma are in need for biologics, therefore this treatment should be managed by specialists with ample experience only

Children with difficult-to-treat and severe asthma: Item 7 R1

Specialist asthma centres and staffing

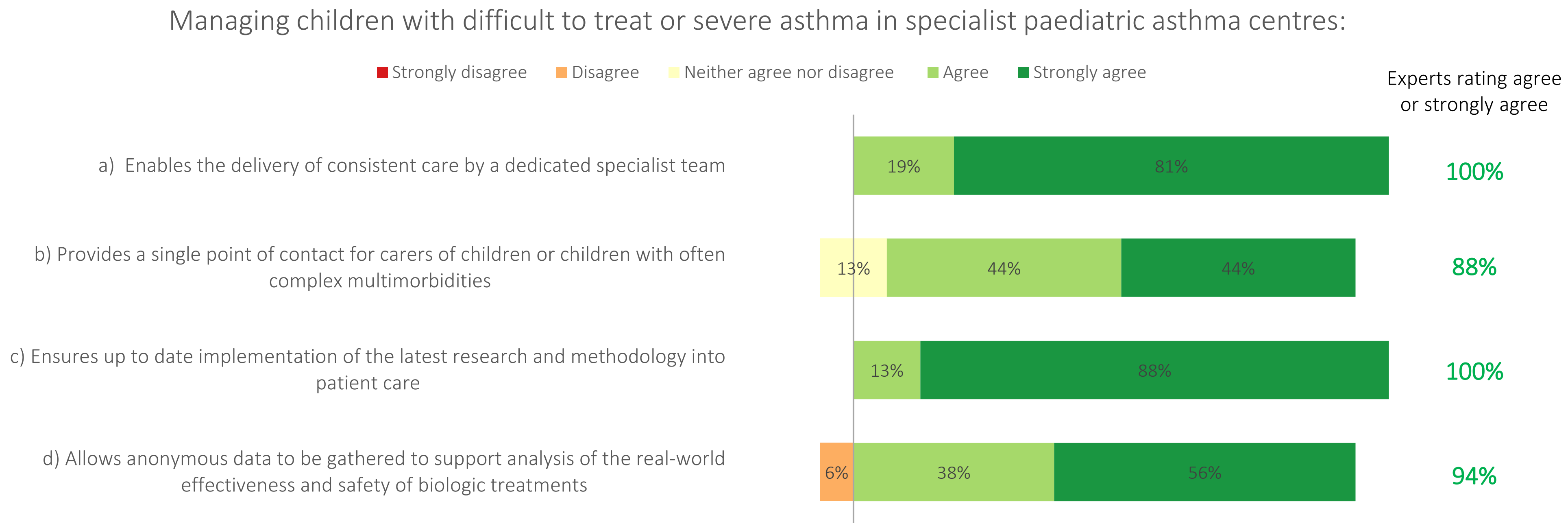

	% Rating Agree or Strongly agree
a)	100
b)	88
c)	100
d)	94

PLEASE ADD INFORMATION TO HELP EXPLAIN YOUR ANSWER

- The strict interpretation of GDPR regulation in my country prohibits us to take part in the SPACE registry trial
- d) due to interpretation of GDPR-law this is difficult

Children with difficult-to-treat and severe asthma: Item 6 R2 (Item 8 R1)

Specialist asthma centres and staffing

The multidisciplinary team members who are essential for specialist paediatric asthma centres are:

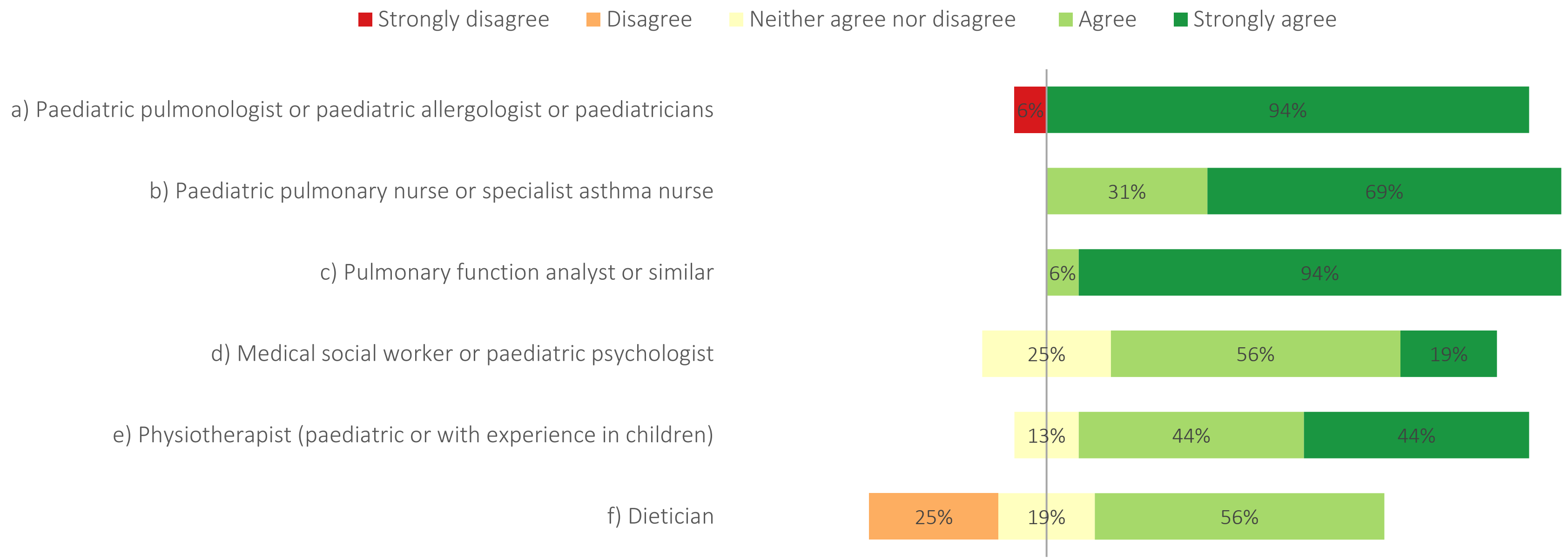

	% rating agree or strongly agree	
	R1	R2
Paediatric pulmonologist or paediatric allergologist or paediatricians*	100	94
Paediatric pulmonary nurse or specialist asthma nurse	100	100
Pulmonary function analyst or similar	100	100
Medical social worker or paediatric psychologist	75	75
Physiotherapist (paediatric or with experience in children)†	81	88
Dietician	75	56

**R1: Paediatric pulmonologist or paediatric allergologist or pulmonologist with a paediatric specialty*
†R1: Paediatric physiotherapist

PLEASE ADD INFORMATION TO HELP EXPLAIN YOUR ANSWER

- f) Dietician on indication: food-allergies, under- or overweight
- for a specialised paediatric asthma center a paediatric pulmonologist is essential. It is not or an allergologist or paediatrican
- It is useful to have a medical social worker or psychologist to help children and families, but not mandatory. The same for dietician (if not available in the paediatric asthma centre, visits with dietician can be organised outside the center)
- a) only pediatrician with pulmonology/allergology subspecialty b) agree but unfortunately financially difficult
- It depends how a "Specialist asthma centre" is defined. Under (a) pediatric pulmonologist, allergologist and pediatrician are individual options. If a specialist paediatric asthma center is regarded as a center where children with severe asthma are treated and evaluated I think you would need somebody in your team who is able to perform bronchoscopy and has experience with the prescription of biologics. In my experience a general pediatrician usually does not have this expertise so I wonder if a pediatrician should be included in this description.
- f) for food allergies yes, for pure, uncomplicated asthma no need

Children with difficult-to-treat and severe asthma: Item 7 R2 (Item 8 R1)
Specialist asthma centres and staffing

Additional specialists who are desirable as members of the extended multidisciplinary team in specialist paediatric asthma centres include:

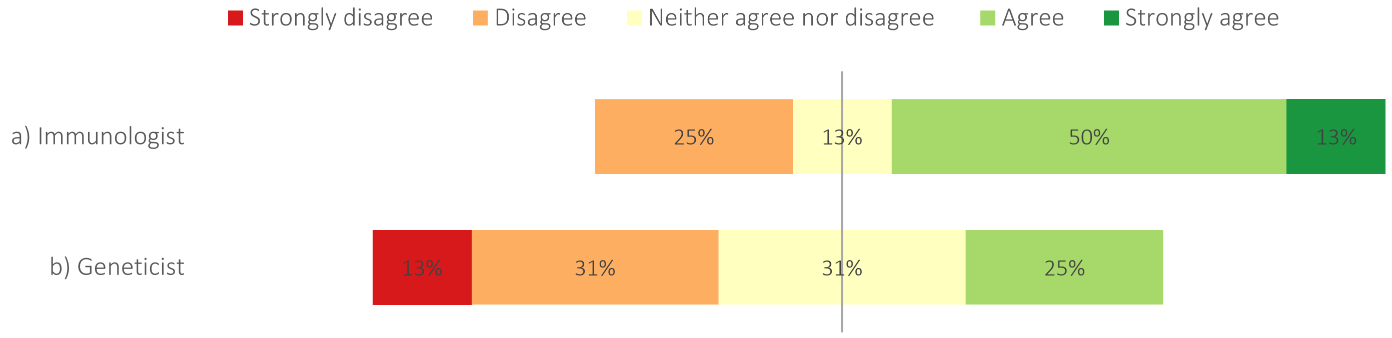

	% rating agree or strongly agree	
	**R1**	**R2**
Immunologist	44	63
Geneticist*	-	25

**Added in R2*

PLEASE ADD INFORMATION TO HELP EXPLAIN YOUR ANSWER
- Both on consultation base
- Both of them are pediatricians desirable as members of the extended multidisciplinary team in specialist paediatric asthma centres but they don't have to be in same centre.

Children with difficult-to-treat and severe asthma: Item 12 R2 (Item 14 R1)

Specialist asthma centres and staffing

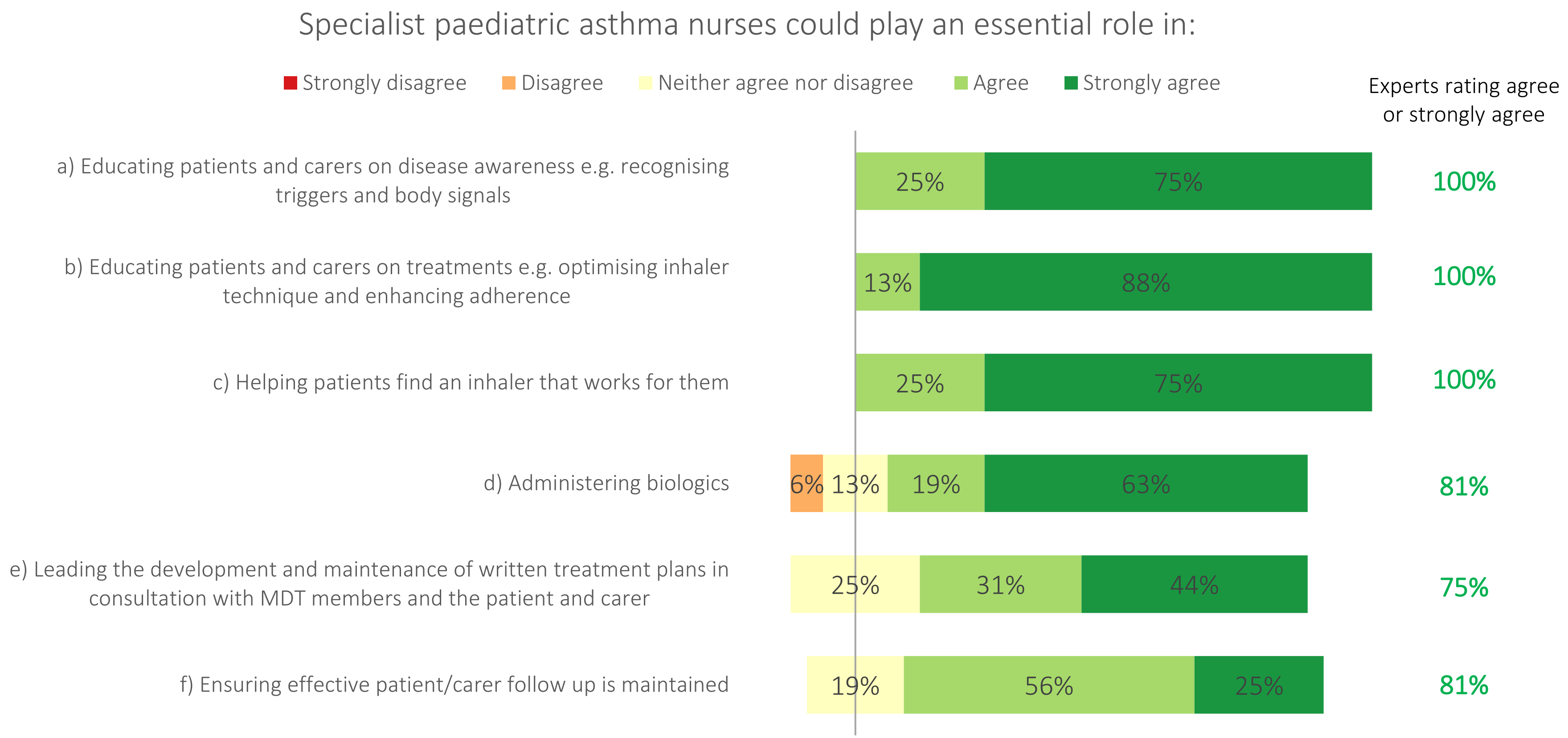

PLEASE ADD INFORMATION TO HELP EXPLAIN YOUR ANSWER

- d can be done by nurses in the daycare unit f) can be done by administrative team
- administering biologics, and leading written treatment plan can be done either by physician or nurse according to each center habits
- Regarding (D) it really depends on the local situation. You don't need a specialist pediatric asthma nurse to administer a biologic, depending on local situations this could be done by any nurse.

Children with difficult-to-treat and severe asthma: Item 8 R2 (Item 9 R1)

Facilities and tests

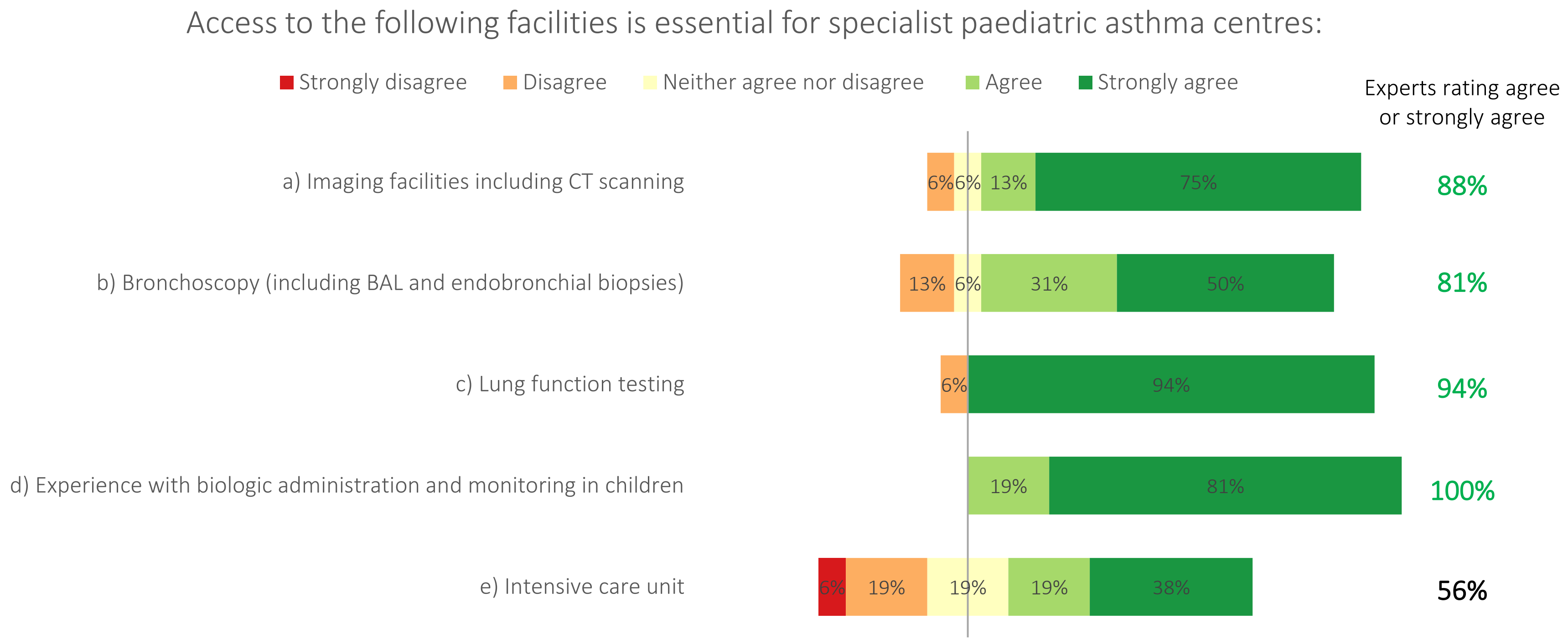

PLEASE ADD INFORMATION TO HELP EXPLAIN YOUR ANSWER

- a+b+c) or a referral center where these tests can be performed. Not necessarily available in the center
- bronchoscopy and BAL can be referred in another center if needed, and children with severe asthma attacks can be referred to another center with ICU

Children with difficult-to-treat and severe asthma: Item 9 R2 (Item 10 R1)

Facilities and tests

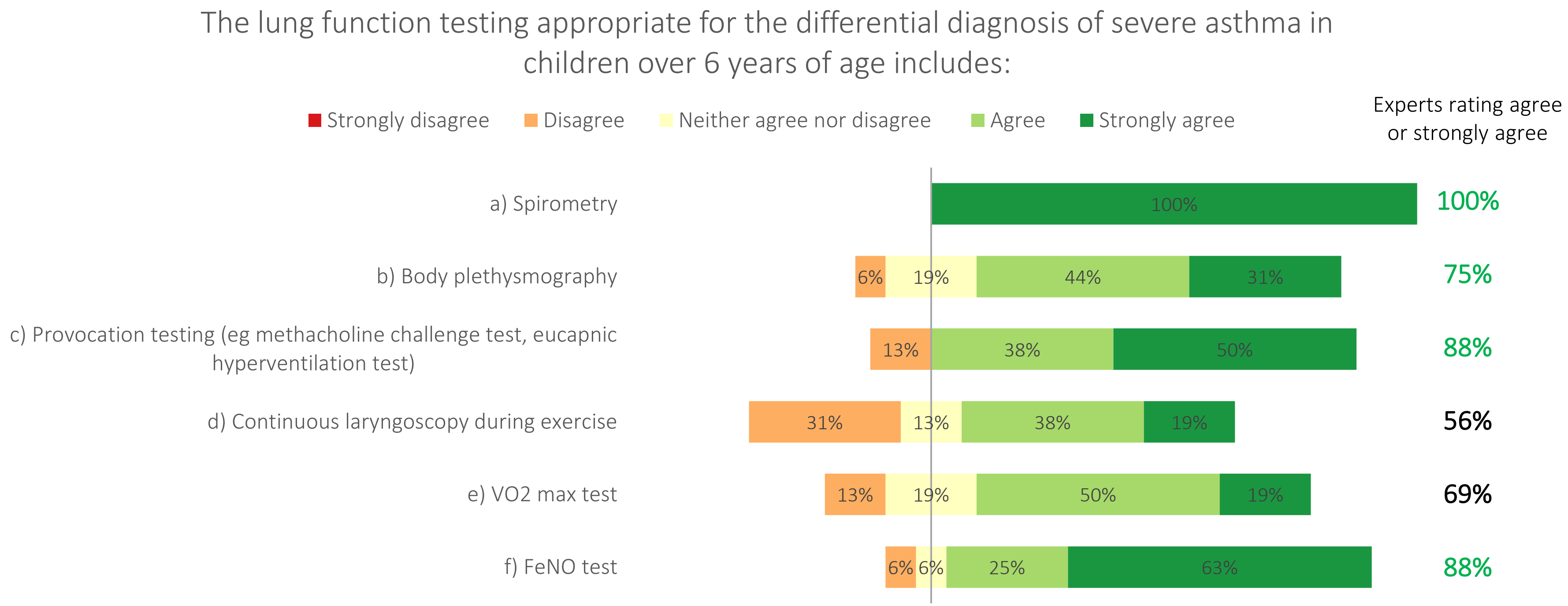

PLEASE ADD INFORMATION TO HELP EXPLAIN YOUR ANSWER

- d) Not for every center
- c) eucapnic hyperventilation test could be removed from the list, d) maybe rephrase the question into lung function tests may include? g) we use FeNo for follow up, not for differential diagnosis
- Continuous laryngoscopy during exercise, and FeNO tests, are useful in some children, but not in all of them. Most centres don't use provocation testing in children. The other tests (spirometry, body plethysmography, and FeNO), are essential in evaluation of severe asthma in children.
- We do methacholine challenge for 12 years or older and EVH for 9-10 years or older

Children with difficult-to-treat and severe asthma: Item 11 R1

Shared decision making and communication

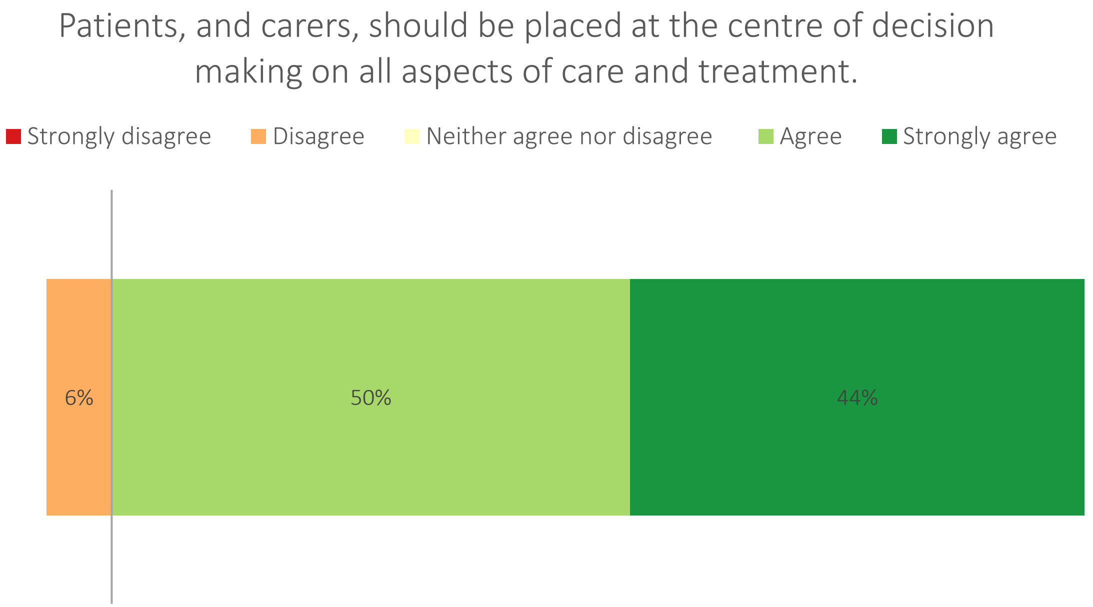

% Rating Agree or Strongly agree
94

PLEASE ADD INFORMATION TO HELP EXPLAIN YOUR ANSWER

- You cannot always place patient/care in the centre of decision
- I feel this is an open door

Children with difficult-to-treat and severe asthma: Item 12 R1

Shared decision making and communication

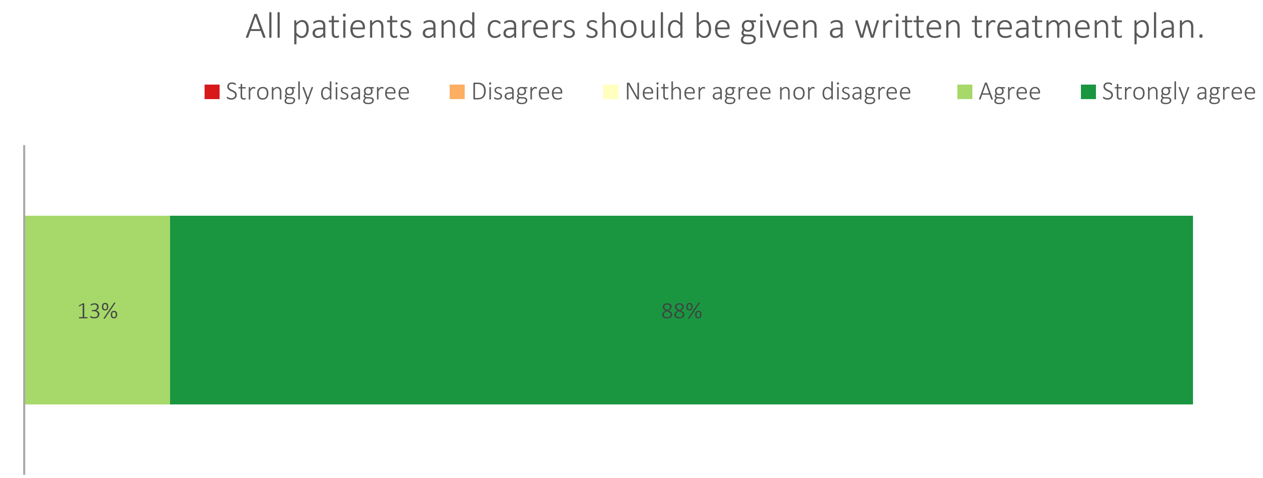

% Rating Agree or Strongly agree
100

PLEASE ADD INFORMATION TO HELP EXPLAIN YOUR ANSWER

- Or a digital one

Children with difficult-to-treat and severe asthma: Item 10 R2 (Item 13 R1)
Shared decision making and communication

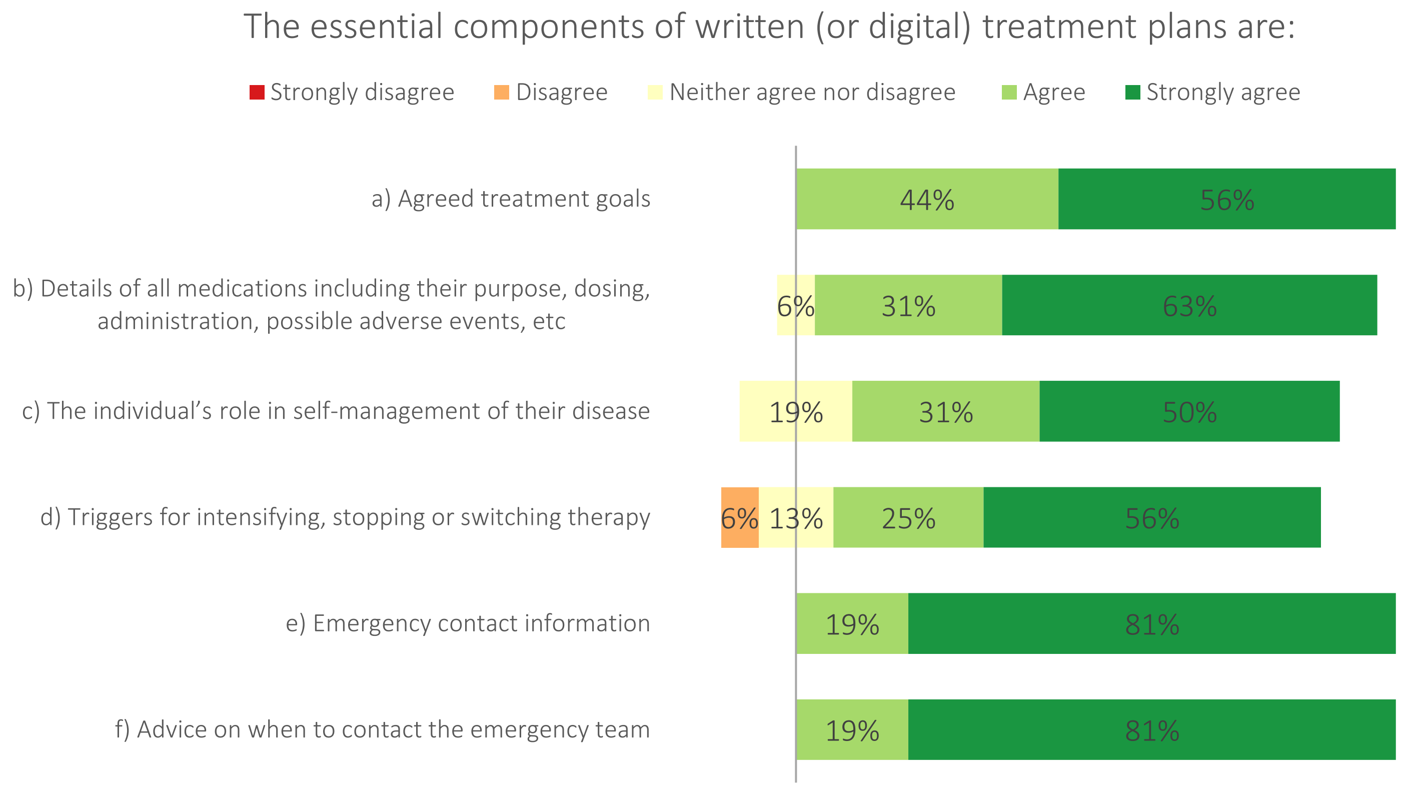

	% rating agree or strongly agree	
	R1	R2
Agreed treatment goals	100	100
Details of all medications including their purpose, dosing, administration, possible adverse events, etc	81	94
The individual's role in self-management of their disease	88	81
Triggers for intensifying, stopping or switching therapy	88	81
Emergency contact information	94	100
Advice on when to contact the emergency team	94	100

PLEASE ADD INFORMATION TO HELP EXPLAIN YOUR ANSWER

- b) except for side effects. c) triggers for intensifying: agree, stopping and switching: depends on what is meant. If it means switch from e.g. budesonide to prednisolone: agree, if it means change, or reducing salbutamol: agree
- dosage of treatment should be in the written treatment plan, the possible adverse events is not mandatory
- b) potential medication side effects should not be prominent in the treatment plan, more as extra info leaflet. the asthma plan should concise and clear c) what is meant by this?
- written plan should not be too complex ; simple plan is important
- On D I of course agree with intensifying the use of for instance SABA, but I don't think I would include something like "switching therapy".
- written treatment plans should be short and concise and easy to understand

Children with difficult-to-treat and severe asthma: Item 11 R2 (Item 13 R1)

Shared decision making and communication

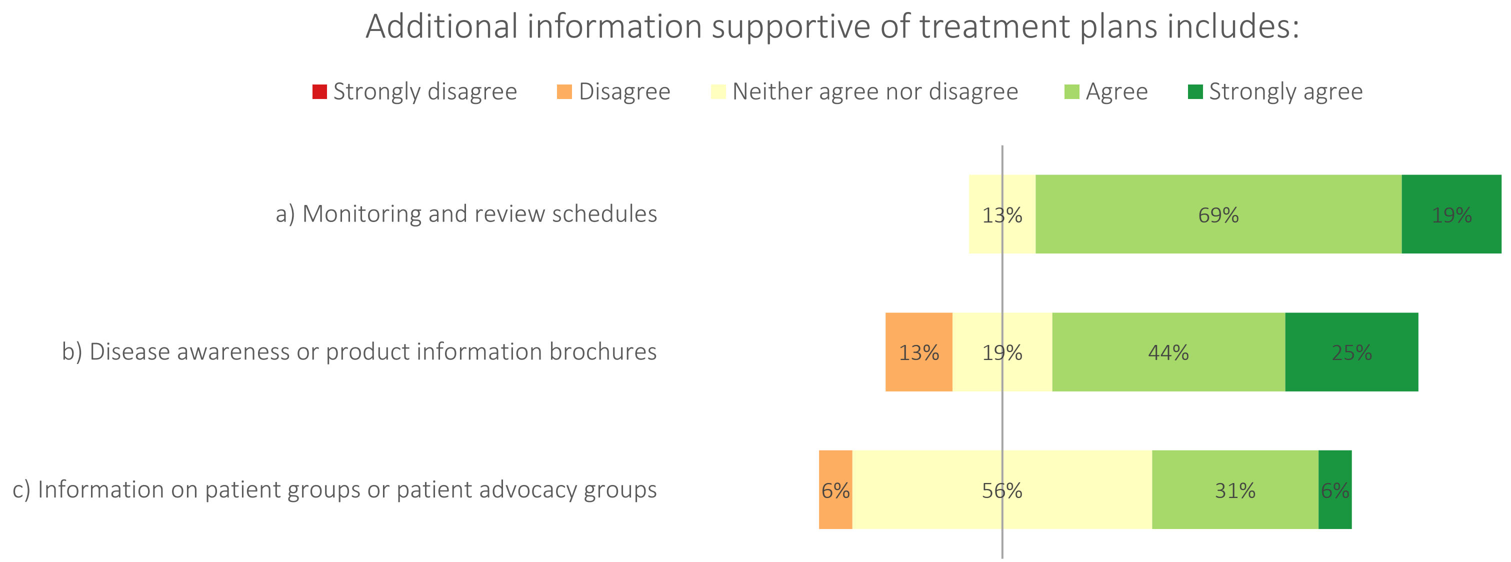

	(% rating agree or strongly agree)	
	**R1**	**R2**
Monitoring and review schedules	63	88
Further disease awareness or product information brochures	38	69
Information on patient groups or patient advocacy groups	25	38

PLEASE ADD INFORMATION TO HELP EXPLAIN YOUR ANSWER

- in a separate brochure or less prominent in the asthma action plan
- not sure what is meant by a. C only of this groups are 'appropriate'

Children with difficult-to-treat and severe asthma: Item 15 R1

Shared decision making and communication

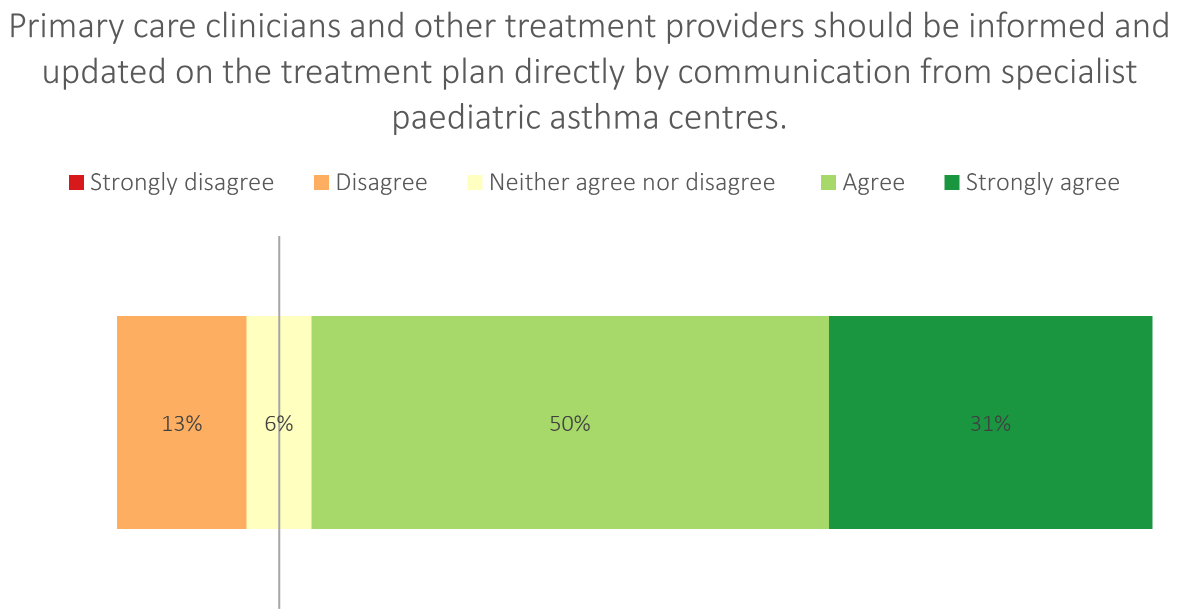

% Rating Agree or Strongly agree
81

PLEASE ADD INFORMATION TO HELP EXPLAIN YOUR ANSWER

- Primary care clinicians should have access to the patient records of specialist asthma centres
- Usually these children would primarily contact and visit their pediatric pulmonologist or perhaps a general paediatrician from a local hospital. So I would say that all treatment providers that are involved in shared care should be updated, but often a primary care clinician is not involved as a treatment provider in this population
- Justn ot possible. They have access to electronic patient report
- GP's are not very permanent in our system. They do not do follow-ups from their side but only based on the patient's activity to book an appointment

Children with difficult-to-treat and severe asthma: Item 16 R1

Shared decision making and communication

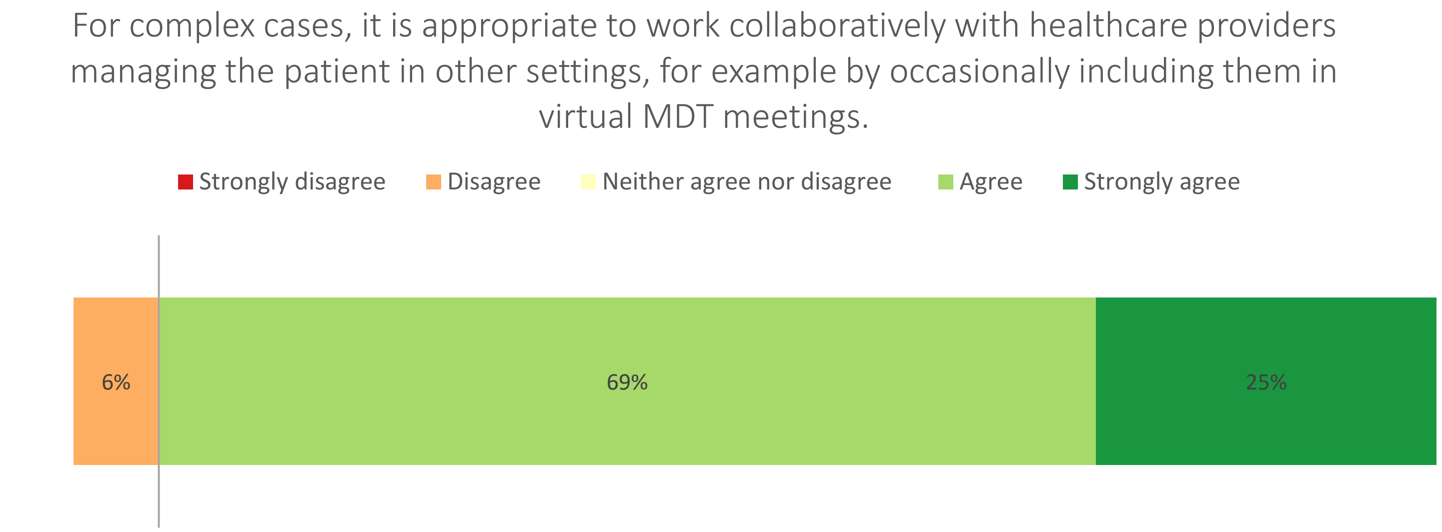

% Rating Agree or Strongly agree
94

PLEASE ADD INFORMATION TO HELP EXPLAIN YOUR ANSWER

- In an optimal system yes but we do not have resources for this

Children with difficult-to-treat and severe asthma: Item 13 R2 (Item 17 R1)

Transition to adult services

The following factors are essential to achieve effective transition from paediatric to adult centres:

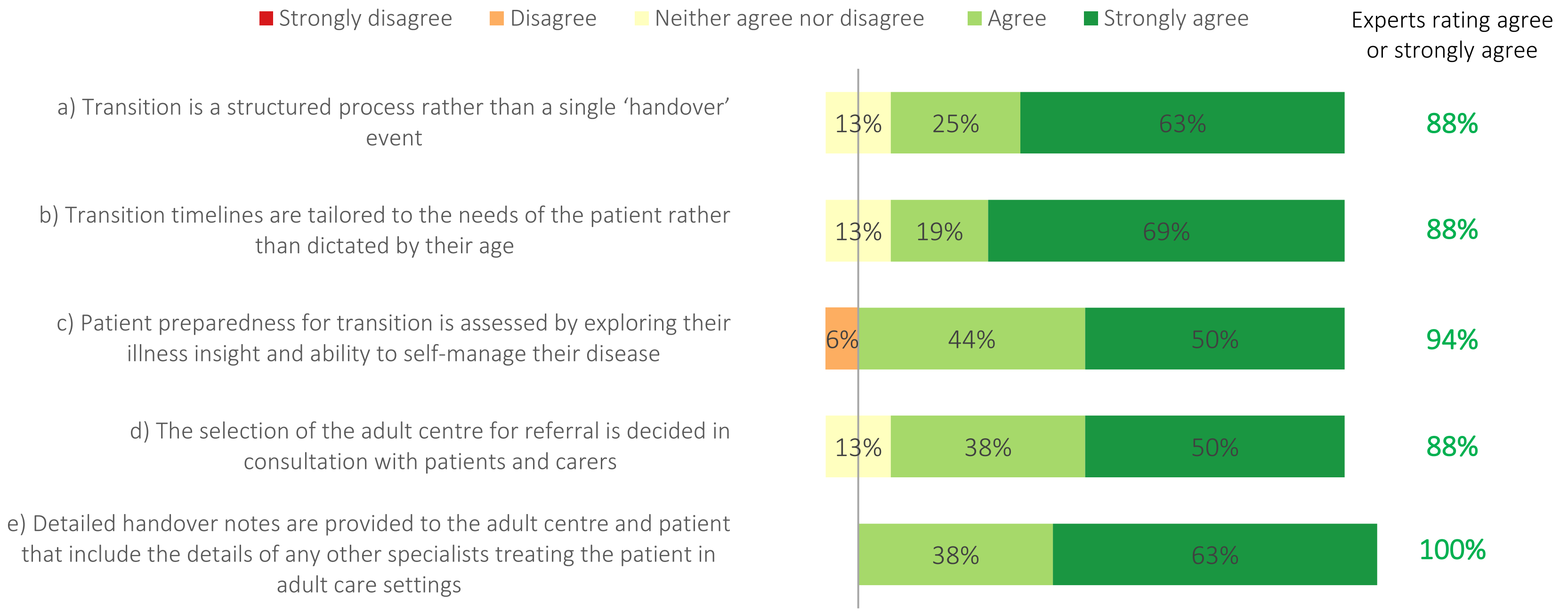

PLEASE ADD INFORMATION TO HELP EXPLAIN YOUR ANSWER

- Some patients don't have the ability to self manage the disease even at adult age, but that should not prevent the transition
- d) resources are also a factor, options might be limited

Children with difficult-to-treat and severe asthma: Item 14 R2 (Item 17 R1)

Transition to adult services

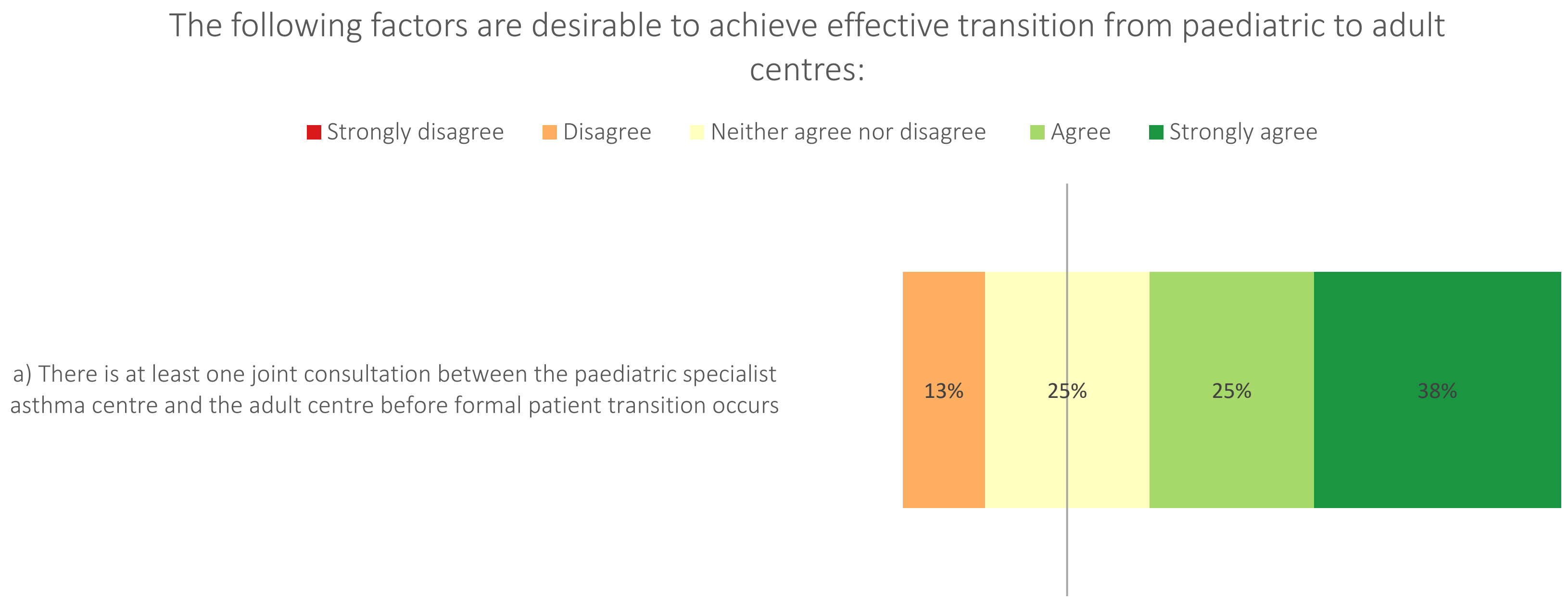

% rating agree or strongly agree	
**R1**	**R2**
56	63

PLEASE ADD INFORMATION TO HELP EXPLAIN YOUR ANSWER

- a) Ideally so, sometimes when patient moves to different city for study eg is hard in daily practice
- depends on the severity of asthma
- the joint consultation helps a lot in lost of care; many children transitioning stop their follow up
- in contrast to complexer chronic diseases, I find transition for asthma not that difficult.

Care related to biologic therapy

Care related to biologic therapy: Item 19 R1

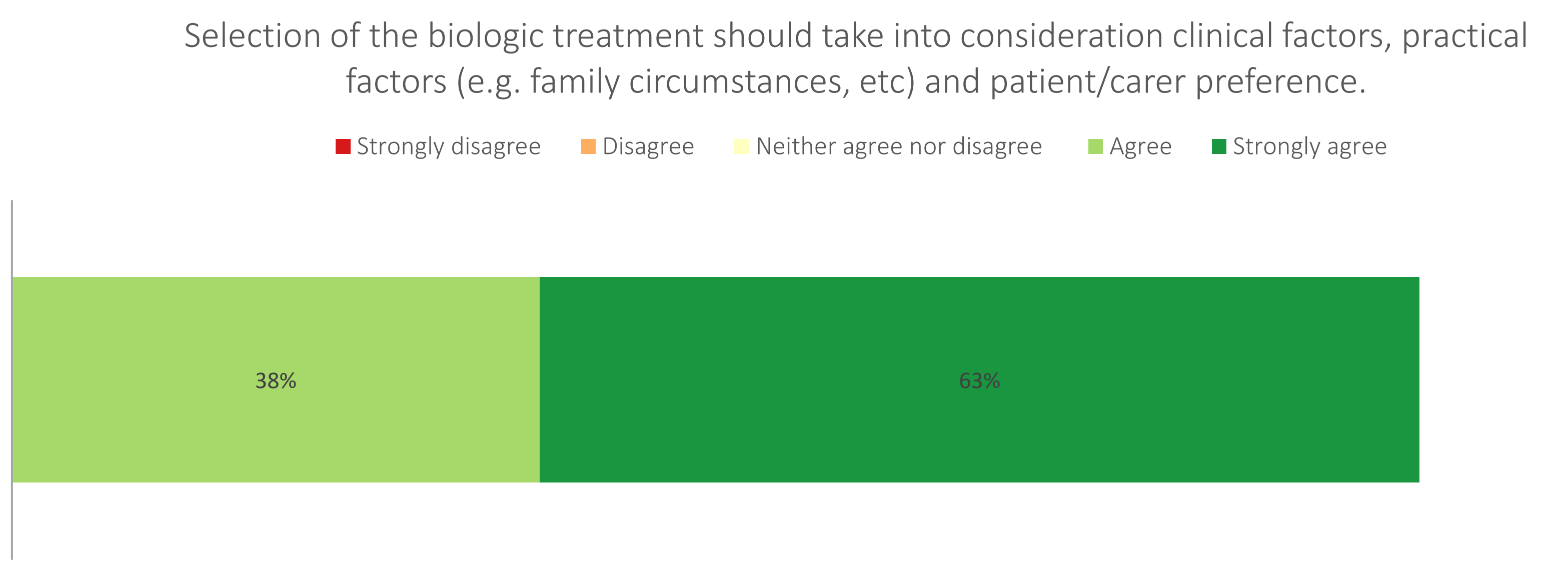

% Rating Agree or Strongly agree
100

PLEASE ADD INFORMATION TO HELP EXPLAIN YOUR ANSWER

- Plus cost and reimbursement

Care related to biologic therapy: Item 15 R2 (Item 20 R1)

Both hospital or homecare delivery of biologic treatment is appropriate, however, homecare with biologics should be avoided under the following conditions:

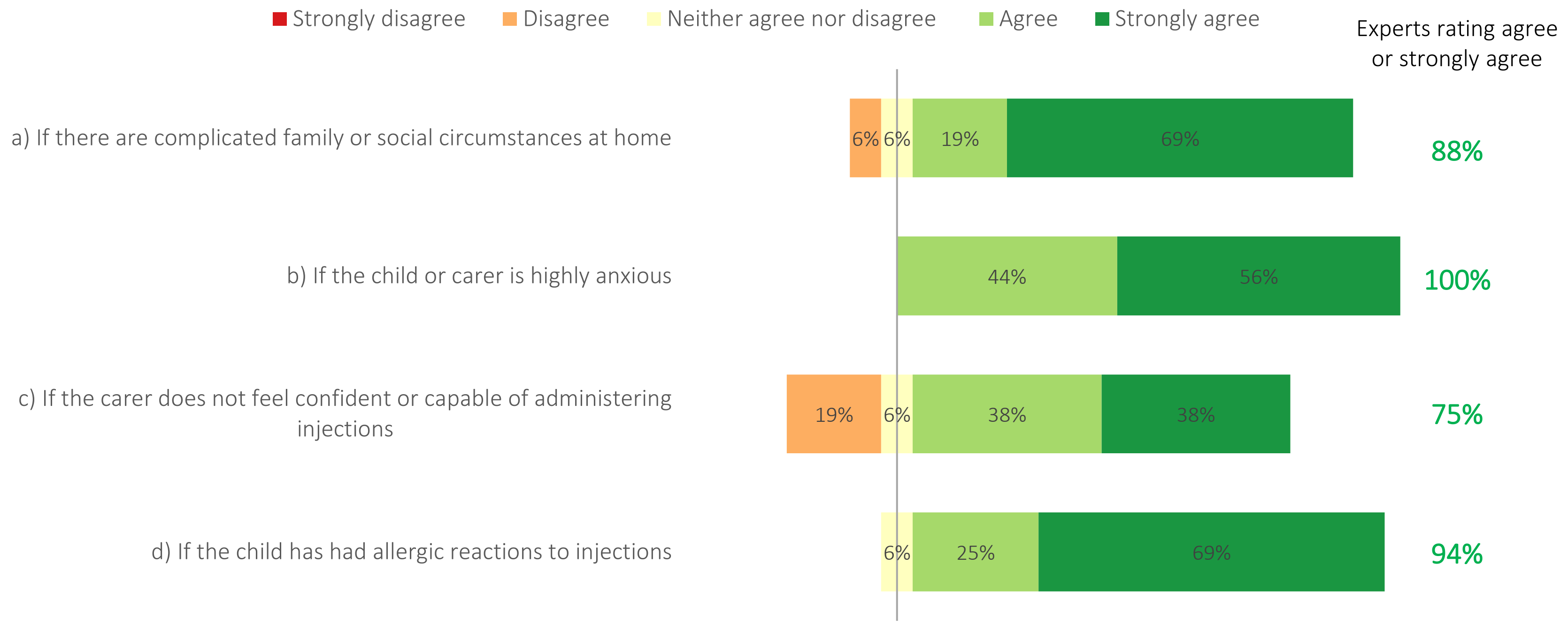

PLEASE ADD INFORMATION TO HELP EXPLAIN YOUR ANSWER

- c) Consider administration by nurse at home or at GP
- d) depends on type of reaction, frequency and whether the reaction has stopped to occur
- c) can be given by homecare
- d) depends on the reaction and the drug
- in a and c a home nurse can be organised
- c) with education they may fell more confident and capable

Care related to biologic therapy: Item 16 R2 (Item 20 R1)

If the child has very poor symptom perception, home spirometry may enable effective homecare delivery of biologic treatment.

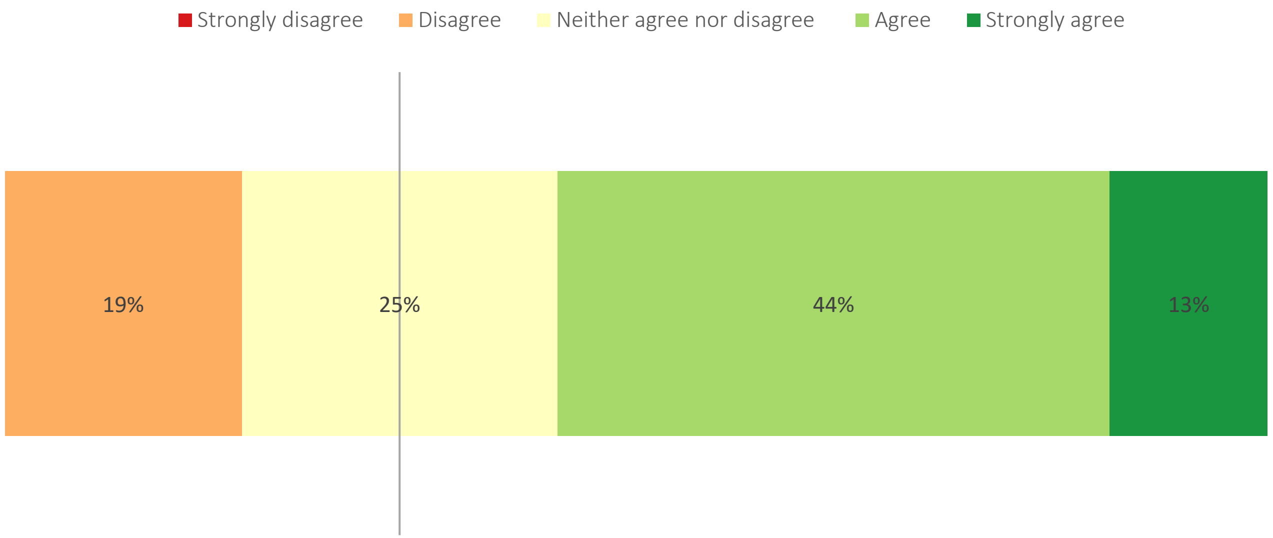

	% rating agree or strongly agree	
	R1	R2
If the child has very poor symptom perception, as spirometry may need to be performed	63	56

This question was originally posed as part of the previous question (Item R1-20). Many comments related to the possibility of home spirometry, so for R2 this was pulled out as a separate question to explore this possibility further.

PLEASE ADD INFORMATION TO HELP EXPLAIN YOUR ANSWER

- I don't get this item. In the phase before start of biologics home spirometry is very important to me in patients with very poor symptom perception for self-management. If despite of home spirometry use asthma is still poorly controlled I would start biologics. I don't understand in what way it may enable treatment with biologics
- home spirometry may not be reliable in children due to lack of cooperation; if the child has very poor symptom perception, delivery of biologic treatment should be performed at the clinical center and not at home
- Why not measure PEF at home?
- I have lots of experience in this - and the home spirometry may both indicate the need for biologics, or that the child actually are totally controlled on their present medication and their perceived symptoms are not due to their lung function
- not too much evidence but does make sense

Care related to biologic therapy: Item 17 R2 (Item 20 R1)

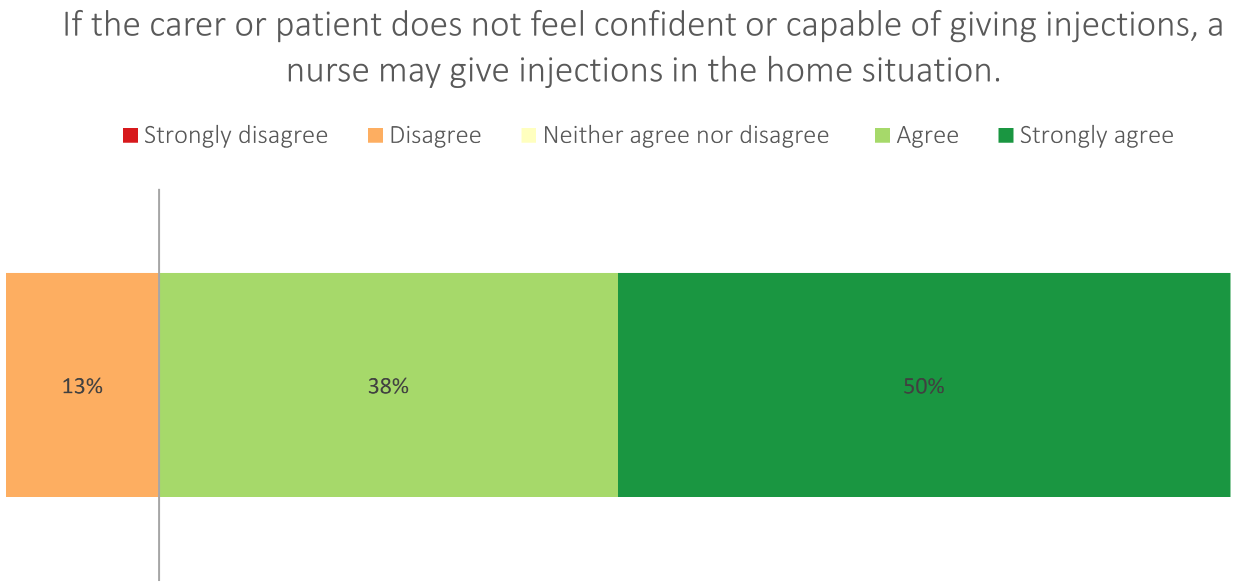

% rating agree or strongly agree	
**R1**	**R2**
63	88

This question was originally posed as part of the previous question (Item R1-20). Several comments related to the possibility of nurses assistance with home delivery of biologics, so this was pulled out as a separate question to explore this possibility further.

PLEASE ADD INFORMATION TO HELP EXPLAIN YOUR ANSWER

- The injections can be given in their nearest health care center
- or nurse can give it at school or primary care setting

Care related to biologic therapy: Item 18 R2 (Item 21 R1)

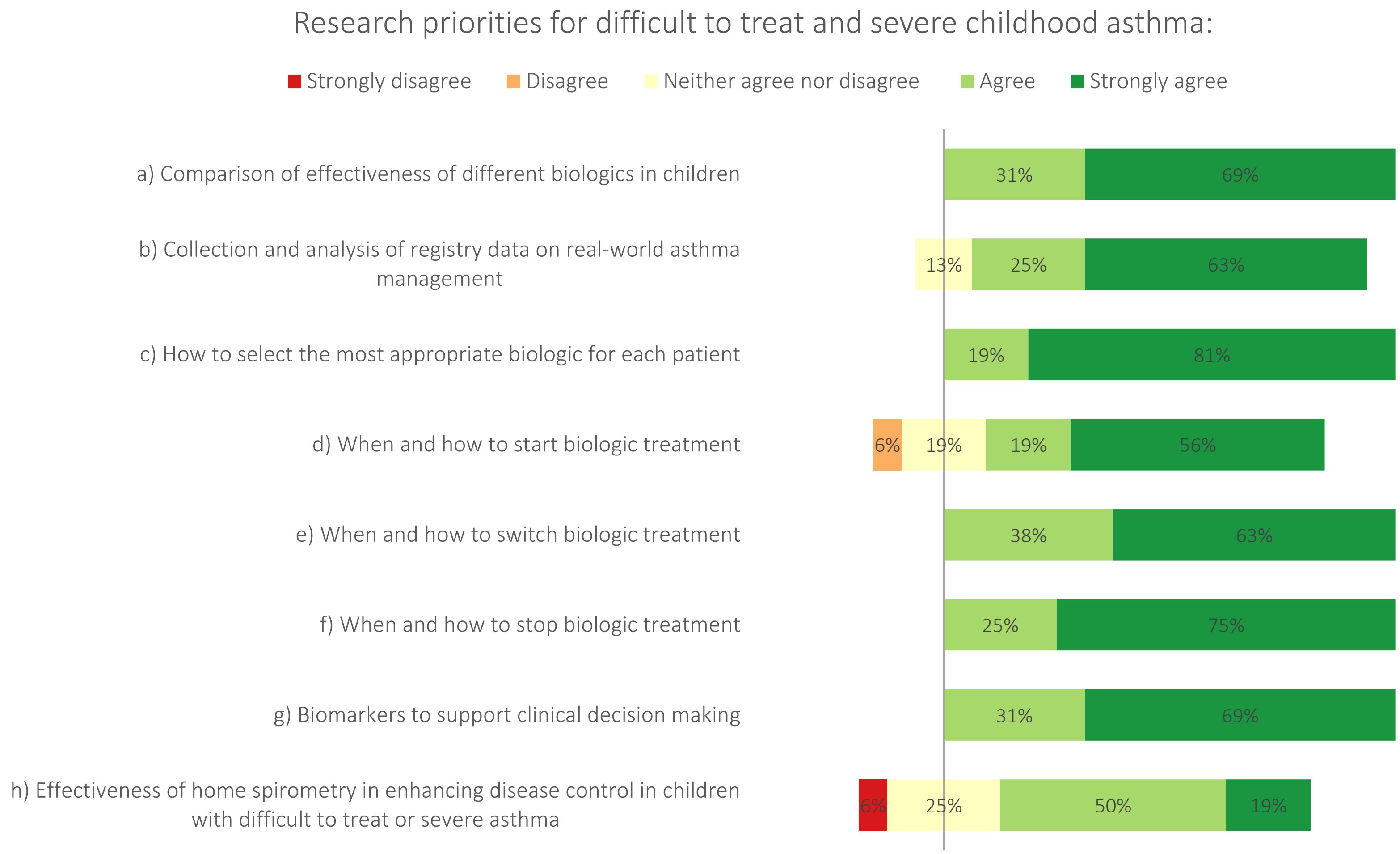

	% rating agree or strongly agree
a) Comparison of effectiveness of different biologics in children	100
b) Collection and analysis of registry data on real-world asthma management	88
c) How to select the most appropriate biologic for each patient	100
d) When and how to start biologic treatment	75
e) When and how to switch biologic treatment	100
f) When and how to stop biologic treatment	100
g) Biomarkers to support clinical decision making	100
h) Effectiveness of home spirometry in enhancing disease control in children with difficult-to-treat or severe asthma	69

This item was created from free text responses in R1

PLEASE ADD INFORMATION TO HELP EXPLAIN YOUR ANSWER

- h) I see effect of this in my daily practice in children with poor perception of asthma symptoms
- first priority would be to have more data in the individual biological in childhood asthma
- Regarding point h - there is already a lot of studies on this - see for example this paper published in ERJ from 2019: Clinical effect on uncontrolled asthma using a novel digital automated self-management solution: a physician-blinded randomised controlled crossover trial: https://erj.ersjournals.com/content/54/5/1900983
